# Non-Invasive Blood Glucose Monitoring Technology: A Review

**DOI:** 10.3390/s20236925

**Published:** 2020-12-04

**Authors:** Liu Tang, Shwu Jen Chang, Ching-Jung Chen, Jen-Tsai Liu

**Affiliations:** 1 Research Center for Materials Science and Opti-Electronic Technology, College of Materials Science and Opti-Electronic Technology, University of Chinese Academy of Sciences, Beijing 100049, China; tangliu18@mails.ucas.ac.cn; 2 Department of Biomedical Engineering, I-Shou University, Kaohsiung City 82445, Taiwan; sjchang@isu.edu.tw; 3 Research Center for Materials Science and Opti-Electronic Technology, School of Opto-Electronic Technology, University of Chinese Academy of Sciences, Beijing 100049, China

**Keywords:** non-invasive, glucose biosensor, optics, microwave, electrochemistry, ISF glucose, reverse iontophoresis

## Abstract

In recent years, with the rise of global diabetes, a growing number of subjects are suffering from pain and infections caused by the invasive nature of mainstream commercial glucose meters. Non-invasive blood glucose monitoring technology has become an international research topic and a new method which could bring relief to a vast number of patients. This paper reviews the research progress and major challenges of non-invasive blood glucose detection technology in recent years, and divides it into three categories: optics, microwave and electrochemistry, based on the detection principle. The technology covers medical, materials, optics, electromagnetic wave, chemistry, biology, computational science and other related fields. The advantages and limitations of non-invasive and invasive technologies as well as electrochemistry and optics in non-invasives are compared horizontally in this paper. In addition, the current research achievements and limitations of non-invasive electrochemical glucose sensing systems in continuous monitoring, point-of-care and clinical settings are highlighted, so as to discuss the development tendency in future research. With the rapid development of wearable technology and transdermal biosensors, non-invasive blood glucose monitoring will become more efficient, affordable, robust, and more competitive on the market.

## 1. Introduction

### 1.1. Overview of Diabetes

Diabetes is one of the most common lifelong chronic diseases in human beings. It is mainly caused by genetic factors, immune disorders and other factors on the human body, leading to islet function decline and insulin resistance, etc., resulting in imbalance of glucose level in the body, which is manifested as failure of glucose metabolism and hyperglycemia [1]. Diabetes is divided into two categories: type 1 and type 2. Deficient secretion of insulin in the pancreas leads to diabetes type 1. Type 2, on the other hand, is mainly due to the ineffective use of insulin caused by the insulin resistance and insulin sensitivity decreasing in patients [2]. Diabetes has a high incidence rate, numerous complications, wide pathogenic factors, difficulty in curing and causes other serious hazards to human health. Therefore, many related fields have been actively devoted to the research of diabetes [3].

More than 90% of people with diabetes are type 2, and the International Diabetes Federation estimates that the proportion of the world’s population affected by diabetes will rise sharply and the number of diabetics will continuously increase [4]. According to the World Health Organization (WHO), currently there are around 450 million cases of diabetes in the world, and the number could potentially reach 700 million by 2045, with an increase to 39.7 million by 2030 and 60.6 million in 2060 in the United States alone [5]. In addition to the large number of diagnosed patients, there is a significant proportion of the population that is undiagnosed for various reasons or at potentially high risk. Therefore, the prevention of diabetes has been paid more attention in various countries, especially in developed countries, and the diagnosis and treatment of diabetes has become a subject with immense practical significance and economic benefits.

In the human body, glucose provides energy for cell metabolism. In addition to blood, glucose is widely contained in intracellular fluids, interstitial fluids (ISF), tears, saliva and urine [1]. At present, the blood glucose concentration is the main basis for the diagnosis of diabetes. The standard was introduced by the WHO in 2009, setting the fasting blood glucose (FBG) of normal people at 3.9–6.1 mM, and the blood glucose 2 h after a meal at 7.8 mM or less. Patients with typical symptoms of diabetes (polyuria, polydipsia and unexplained weight loss) who have arbitrary blood glucose ≥ 11.1 mM, FBG ≥ 7.0 mM or 2 h after a meal ≥ 11.1 mM, which means diabetes can be diagnosed. Apart from hyperglycemia, hypoglycemia would damage the human body as well. Clinically, hypoglycemia is defined as a condition where the blood glucose concentration is lower than 3.9 mM and lasts for more than 5 min, which is a common complication of insulin therapy or oral hypoglycemic agents [6]. For elderly patients in particular, the risk coefficient of hypoglycemia is even higher. The incidence of hypoglycemia at night is relatively high, and it is difficult to monitor it in a timely way with traditional blood glucose detection methods. Strict blood glucose control is also likely to increase the risk of hypoglycemia [7]. Therefore, continuous glucose monitoring (CGM) in diabetics may be of more clinical application value and more in line with market trends.

### 1.2. Methods for Monitoring Blood Glucose Concentration

According to whether the blood glucose test has caused injury to human skin, it can be simply divided into invasive and non-invasive blood glucose monitoring.

#### 1.2.1. Invasive Blood Glucose Monitoring

At present, invasive blood glucose detection technology is mainstream, convenient and practical, so both hospitals and household glucometers adopt the method of blood sampling first and then analyzing it in vitro for blood glucose measurement. In hospitals, the blood drawn from the subjects on an empty stomach in the morning, and the blood glucose concentration is accurately measured by automatic biochemical analyzer. Although the results of this method are precise and can be used as an important basis for the diagnosis of diabetes, it is unfit for continuous monitoring of diabetics due to its tedious process, long detection time and large amount of venous blood extraction.

Self-monitoring of blood glucose (SMBG) refers to the monitoring of blood glucose concentration at a specific point in time [8], which is usually done with a home electronic glucose meter. However, household blood glucose monitors typically use glucose oxidase biosensors, collect fingertip blood with a disposable strip of paper, and determine the concentration of blood glucose through the chemical reaction current of the strip. At present, the most common commercial glycemic meter brands on the market are Roche, Sano, Omron, Johnson and Johnson, Bayer, Abbott, Echeng, Ecco and so on. The salient advantages with portable, cheap, simple to operate, relatively accurate data and monitoring multiple times a day are the reason the commercial glucose meter is widely used in the home. However, its disadvantages are also obvious. Since a certain amount of blood is taken from the fingertips before each test, the skin will be punctured deeply. With the increase in the frequency of blood collection, it is difficult for patients’ fingertip wounds to heal in time. Not only does it expend the risk of external environmental infection, but also the patient will suffer great pain and stress before the daily blood collection. On the other hand, both the test paper and the blood needle are disposable consumables. If they are used frequently, it will cost a lot for families in underdeveloped countries and regions. Moreover, the shelf life of the strip is not long, and improper storage will interfere with the accuracy of blood glucose detection value.

Healthcare guidelines recommend that SMBG should be administered four times a day, increasing to 10 times a day during illness or poor control [9]. However, some reports indicate that about 40% to 50% of diabetics do not follow the guidelines [10]. Failure to inject insulin on time may lead to serious diabetes complications such as diabetic ketoacidosis (DKA), cardiovascular diseases, blindness, stroke and neurasthenia [6,11]. Moreover, excessive insulin treatment could cause a sudden and drastic drop in blood glucose, which may bring grave consequences about seizures, coma, and even death [2]. Obviously, the current SMBG method makes patients feel uncomfortable and needs to be improved.

#### 1.2.2. Non-Invasive Blood Glucose Monitoring

**Figure 1:**
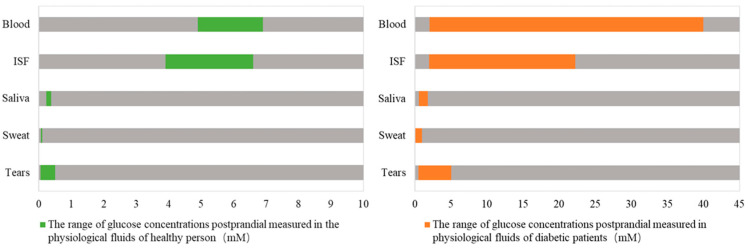
Contrast of glucose concentrations in different physiological fluids between healthy and diabetic people.

Non-invasive blood glucose monitoring, as its name implies, refers to the detection of human blood glucose without causing damage to human tissues. There are lots of methods for non-invasive blood glucose detection, which can be generally divided into optical methods, microwave methods and electrochemical methods. Optical methods include near-infrared reflectance spectroscopy (NIRS), polarized optical rotation, Raman spectroscopy, fluorescence, optical coherence tomography (OCT) and so on [4,12]. As shown in Figure 1 [1], in addition to glucose in human blood, there are also considerable amounts of glucose in other biofluids (such as saliva, tears, sweat, and ISF). Utilizing the coherent correlation between biofluids and blood glucose value, the electrochemical method usually measures the glucose content in body fluids first and obtains the blood glucose value indirectly after the calibration of the algorithm or data model. The range of glucose in ISF is the closest to the range of blood glucose in both healthy and diabetic, which provides a theoretical basis for the development of an ISF glucose sensor. However, a large number of research works have shown that there is a time-lag phenomenon between the ISF glucose and blood glucose, that is, ISF reflects the change of blood glucose level with a certain delay, which ranges from about 4–10 min [13,14,15,16]. Transdermal biofluid extraction generally adopts reverse iontophoresis (RI) technology, which can achieve the purpose of rapid extraction of ISF. The specific method and content are introduced in detail below.

Frequent and regular blood glucose testing is crucial for diabetics, who need regular insulin injections to maintain their blood glucose balance. Therefore, the development and breakthrough of the new painless and stress-free non-invasive blood glucose monitoring technology and precise closed-loop drug delivery system can directly benefit hundreds of millions of patients by avoiding the pain of blood collection, which is very promising and practical research at present.

## 2. Principle and Development of Glucose Biosensors

Since Clark and Lyons first proposed the concept of glucose oxidase (GOx) electrode in 1962 [17], researchers in various countries have made intensive studies and unremitting efforts in electrode materials, electrode construction, electrode coating, enzyme-curing methods etc. At present, according to the different electron transfer mechanisms, the development of electrochemical glucose biosensors (GBs) can be roughly divided into three stages: the classic GOx electrode sensor, the mediator GOx electrode sensor, and the direct GOx electrode sensor.

### 2.1. First-Generation Glucose Biosensors—Classic GOx Electrode

The first-generation of classic GOx electrode sensor indicated the amount of glucose consumed by detecting oxygen consumed or hydrogen peroxide produced in the electrode reaction.

(1) $$\mathrm{Glucose}\left(C_{6}H_{12}O_{6}\right)+O_{2}\overset{\mathrm{GOx}}{\to }\mathrm{Gluconolactone}\left(C_{6}H_{10}O_{6}\right)+H_{2}O_{2}$$

(2) $$H_{2}O_{2}\to 2H^{+}+O_{2}+2e^{-}$$

When hydrogen peroxide is detected, a high potential (more than 1 V) is applied to the electrode anode, and a platinum electrode is usually used as the anode. However, some reductive molecules (such as ascorbic acid, acetaminophen, uric acid, and lactic acid) are readily oxidized at this higher potential [18]. In the early stage, the platinum electrodes were directly in contact with the reaction solution, so there were many side reactions and interference. In general, there are several ways to reduce the interference. One is to cover the electrode with a permselective polymer membrane to avoid the interferant from participating in the reaction on the electrode surface. The second is to reduce the production of other oxidation reactions by reducing the overpotential. For example, Prussian blue (PB) is a very effective electrocatalytic medium, which can reduce the reaction potential of Ag/AgCl electrode to nearly 0 V, thus the specificity and sensitivity of the sensor could be improved. In addition, it is also an effective way to focus on the fact that the electrode performance is enhanced by improving the curing mode and activity of GOx. In addition, the fluctuation of oxygen content will also cause great interference to the sensor, including changes in sensor response and the linear range of detection [18,19,20,21]. Li et al. designed a low-invasive subcutaneous electrochemical glucose biosensor, which was optimized for some defects of the first-generation glucose biosensors [22]. First, the surfactant Triton x-100 was used to electrodeposit multilayer GOx on the electrode for high-density enzyme immobilization. Then it was electrically polymerized with o-phenylenediamine to form a functional coating and permselective polymer membrane. These two steps, on the one hand, strengthened the solidification of the enzyme and improved the performance of the electrode; on the other hand, the electrode surface was prevented from being disturbed by other electroactive compounds in human biofluids, which improved the accuracy. The detection limit of the low-invasive sensor was 0.11 mM, and the detection range of glucose concentration could reach 21 mM. The sensitivity was 2.55 ${\mu \mathrm{AmM}}^{-1}{\mathrm{cm}}^{-2}$ and the linear coefficient was 0.9986. Although there was a trend related to commercial blood glucose meters in practical testing, there were also defects of high delay and low response strength. In general, the first-generation glucose sensors are relatively simple in principle and production, but their sensitivity, detection range and accuracy are not satisfactory owing to the influence of high detection voltage and oxygen dependence.

### 2.2. Second-Generation Glucose Biosensors—Mediator GOx Electrode

**Figure 2:**
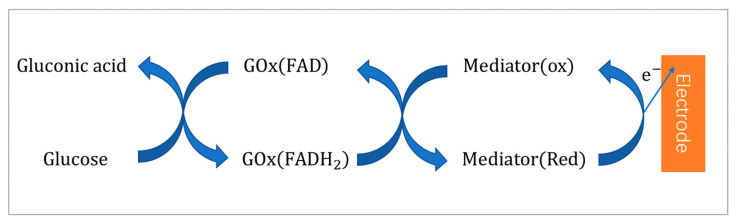
Schematic diagram of the second-generation glucose biosensor.

The second-generation mediator GOx electrode sensors are mainly use redox mediators which can interact directly with the enzyme. The artificial electron acceptors replace oxygen and passes electrons directly between the FAD center of the GOx and the surface of the electrode. The electron transfer mechanism is as follows [20,23]:

(3) $$\mathrm{glucose}+\mathrm{GOD}\left(\mathrm{FAD}\right)\to \mathrm{gluconicacid}+\mathrm{GOD}\left({\mathrm{FADH}}_{2}\right)$$

(4) $$\mathrm{GOD}\left({\mathrm{FADH}}_{2}\right)+2M\left(\mathrm{ox}\right)\to \mathrm{GOD}\left(\mathrm{FAD}\right)+2M\left(\mathrm{red}\right)+2H^{+}$$

(5) $$2M\left(\mathrm{red}\right)\to 2M\left(\mathrm{ox}\right)+2e^{-}$$

where M(ox) and M(red) are the oxidized and reduced forms of the mediator. Figure 2 shows the mechanism of the reaction. In the absence of oxygen to participate in the reaction, the mediator (red) is re-oxidized on the electrode surface and generates the current signal. Common electronic media include ferrocene-based polymers and derivatives (FBPDs), ferricyanide (FIC), hydroquinone (HQ) and conductive organic salts (such as tetrathiafulvalene (TTF), tetracyanoquinodimethane (TCNQ), etc.) [20,24,25]. Among them, FBPDs have the advantages of excellent redox performance, easy of modification, stability, large specific surface area and good electrical conductivity. In recent years, many excellent FBPDs have been widely studied and reported, such as ferrocene with metal complexes (ZnO), conducting polymers (polypyrroles and polythiophenes), nanoparticles (gold and platinum), natural polymers (cellulose and chitosan) and carbon nano tubes [25,26]. Compared with ferrocene and HQ, the FIC mediator dissolves faster and more completely in water, so it may achieve higher sensitivity, wider linear range and shorter response time. Lin et al. designed a second-generation glucose biosensor using FIC. Due to the mutual attraction between positive and negative charges, they used positively charged α-poly-L-lysine (αPLL) as the entrapment matrix for the better immobilization of negatively charged GOx and FIC. The screen-printed strip GBs had good linearity from 2.8 mM to 27.5 mM and a detection limit of 2.3 mM [24]. However, ferrocene or ferricyanide derivatives are generally at risk of permeation and biotoxicity and are not recommended for use in vivo devices [18]. In summary, the second-generation glucose biosensors have a greater current response degree, a shorter response time, higher sensitivity and wider application range than the first generation. With the development of screen-printing technology, it will remain the mainstream of current research.

### 2.3. Third-Generation Glucose Biosensors—Direct GOx Electrode

The presence of oxygen and an electronic mediator will always affect the selectivity and sensitivity of glucose biosensors. The third-generation glucose biosensors aim to use the direct electron transfer between GOx and electrode for detection without the presence of a medium, so as to achieve faster response speed and higher sensitivity. To achieve this, FAD (flavin adenine dinucleotide), the redox center of this glucose oxidase, needs to be closer to the electrode surface to make direct electron transfer more efficient. However, direct electron transfer is difficult because the FAD is coated with a thick layer of protein. Typically, third-generation glucose biosensors use engineered enzymes modified to facilitate chimerism between the enzyme and the electrode; or the enzyme is fixed to a porous polymer electrode. Barathi et al. reported a third-generation glucose biosensor that immobilized GOx directly on electrodes modified by chitosan-supported mesoporous carbon (MPC-CHT) nanocomposite [27]. Due to the enhancement of intermolecular interaction between the CHT and GOx molecule, the electron transfer on the electrode surface is accelerated, so the electrode modified by MPC-CHT-GOx showed excellent electrocatalytic activity. It showed a good linear response over the glucose concentration ranges from 250 μM to 3 mM with a limit of detection (LOD) of 4.1 μM and sensitivity of 56.12 ${\mu \mathrm{AmM}}^{-1}{\mathrm{cm}}^{-2}$. Although the sensitivity has been greatly improved compared with the first generation, the linear detection range was still very narrow (the FBG of healthy people is about 3.9–6.1 mM). With the development of nanotechnology, the nano-size effect of nanomaterials and other nano-effects have been found to effectively promote direct electron transfer between them. Nanostructured electrodes (such as nano-gold electrode, graphene or carbon nano tubes (CNTs) electrode, etc.) coupled with GOx can play a better role in fixing the enzyme layer, which has been studied more. But at present, the detection limit of the third-generation glucose biosensors are not excellent and the linear range is narrow, which cannot meet the ideal requirements. Limited by nanomaterials fabrication technology and cost, it is still in the laboratory research stage.

Over the past 60 years, after three leaps and bounds, the intrinsic principles of electrochemical glucose sensors have been enriched and their outward performance has been greatly improved. Numerous technological innovations (including material, structural, and electrochemical analysis) have opened the door to a wide range of electrochemical glucose biosensors, making glucose sensors highly successful not only in invasive systems, but also in an increasing number of applications in non-invasive monitoring systems. In terms of enzymes, in addition to the common GOx-based [15,28,29], glucose sensors such as glucose dehydrogenase(GDH)-based [14,30], GOx/horseradish peroxidase (HRP) dual enzyme systems [31], β-hydroxybutyrate dehydrogenase (HBD) [11], and non-enzymes [32] have also been heavily researched and developed. The application of new materials, such as porous materials, nanomaterials, and electrocatalytic materials, plays a good role in enhancing the effect of immobilized enzymes, reducing the influence of external environment, and increasing the electron transfer rate, which further improve the sensor stability, sensitivity, and response time. Despite impressive advances in the development of glucose biosensors, the prospect of tight control of diabetes has yet to be realized, especially for non-invasive glucose sensors, which still have room for improvement in immobilizing enzymes, increasing sensitivity, and enhancing long-term stability.

## 3. Non-Invasive Blood Glucose Monitoring Technology—Optical Methods

### 3.1. Near-Infrared (NIR) and Mid-Infrared (MIR) Spectroscopy

The absorption of light by human tissues is closely related to its wavelength. Ultraviolet light may be absorbed easily by DNA and proteins, visible light by hemoglobin, and infrared by water. Since none of these wavelengths can penetrate the human body in large numbers, it is difficult to obtain information about the human body from spectral data. However, near-infrared (NIR, 680–2500 nm) and mid-infrared (MIR, 2500–25,000 nm) have relatively ideal detection spectra. In the near-infrared spectral range, light has a relatively strong ability to penetrate biofluids and soft tissues (>0.5 mm), scatters less than ultraviolet (UV) or visible light, and its sensing and measurement can be achieved by both reflection and transmission [33]. However, MIR-based detection methods can only be implemented in reflective mode, due to the much weaker ability of MIR light to penetrate tissues (a few micrometers) [34,35]. The Beer–Lambert law (Equation (1)) [36,37] provides a mathematical formulation of the method that allows the calculation of absorbance of a sample from the concentration and the thickness [38]:

(6) $$I=I_{0}10^{\left(-l.\varepsilon .c\right)}=I_{0}e^{\left(-l.\mu_{a}\right)}$$

where I is the light intensity at any depth within the absorption medium (W/cm^2^), $I_{0}$ is the initial light intensity (W/cm^2^), l is the absorption depth within the medium (cm), ε is the molar extinction coefficient or molar attenuation coefficient in L/(mmol cm), which depends on the wavelength of incident light and the structure of the absorbing molecules, c is the concentration of absorbing molecules (mmol/L). The product of ε and c is proportional to the absorption coefficient ($\mu_{a}$).

The model shows the reflected/transmitted light intensity as a function of sample thickness, concentration and absorption coefficient, where the effect of scattered light is neglected. Absorbance is defined as $\log(I_{0}/I)$ [37]. In aqueous glucose solutions, NIR/MIR absorption spectroscopy is able to measure the wavelength dependence of the absorbance of glucose. Water is the most abundant substance in biofluids, so the absorption of incident light by water must be considered. In the NIR range, water has two absorption peaks: one between 1350 and 1520 nm and the other between 1790 and 2000 nm. Therefore, the general NIR wavelength windows are 700–1100 nm, 1500–1850 nm and 2000–2400 nm, all of which can be used to measure glucose [39,40,41]. In contrast to these three intervals, water has a lower light absorption in the shorter wavelength range, so the use of shorter wavelengths allows NIR spectroscopy to be more selective by minimizing the interfering effects of water [33,42]. The absorption peak of glucose in the MIR range is between 6250 and 11,110 nm. Apparently, the absorption of light by water is more significant in the MIR range compared to the NIR [35,43,44].

**Figure 3:**
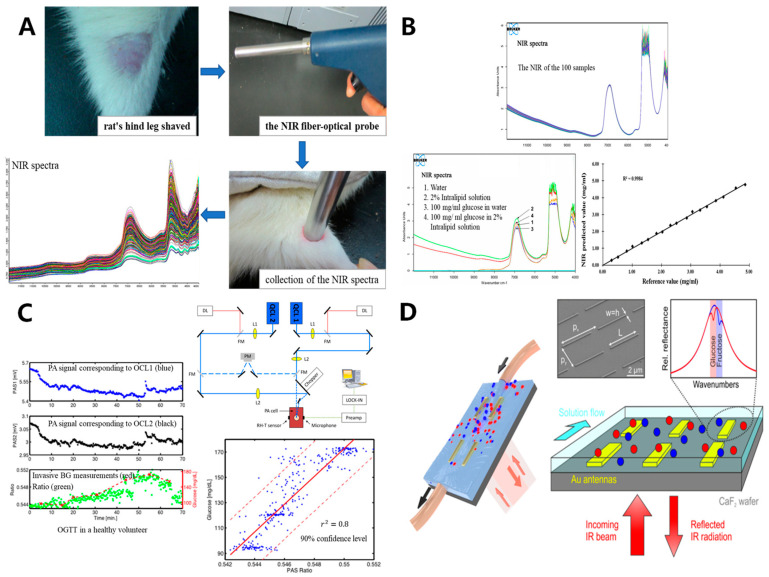
Near-infrared (NIR) and mid-infrared (MIR) spectroscopy methods. (**A**) Noninvasive and fast measurement of blood glucose in vivo by NIR spectroscopy. Adapted with permission [46]. Copyright 2017, Elsevier. (**B**) Rapid and non-destructive measurement of glucose in a skin tissue phantom by near-infrared spectroscopy. Adapted with permission [47]. Copyright 2018, Elsevier. (**C**) Mid-infrared photoacoustic detection of glucose in human skin: towards non-invasive diagnostics. Adapted with permission [35]. Copyright 2016, MDPI. (**D**) Vibrational sensing using infrared nanoantennas: toward the non-invasive quantitation of physiological levels of glucose and fructose. Adapted with permission [48]. Copyright 2019, American Chemical Society.

However, the complexity of the internal structure of human tissues and the superposition of spectral information of various substances will greatly interfere with the accuracy of the results, so it generally requires some algorithm processing to extract the required material information [45]. Xue et al. established two different multivariate calibration models by using partial least squares (PLS) and artificial neural networks (ANN), and concluded that the PLS model had better performance through comparative analysis, with lower root mean square error of validation (RMSEP) of 0.419 and higher correlation coefficients (R) of 96.22% (Figure 3A) [46]. Recently, the RMSEP was reduced to 0.061, and the correlation coefficient R was increased to 99.33% by the team again (Figure 3B) [47]. Nonetheless, this experiment only tested the simulated tissue, and the accuracy of the model may be uncertain when different sample populations (such as age, skin color, gender, skin diseases, etc.) are different, which needs to be further explained. Kottmann et al. [35] proposed two MIR quantum cascade laser (QCL) photoacoustic measurement schemes for human tissues. One is fiber-coupled photoacoustic (PA) cells and tunable QCL, and the other is a dual QCL–PA setup. The device, which indirectly reflects the blood glucose level at the measurement site by testing the glucose level in the ISF, allows for 90 min of continuous measurement but with a 15 min lag time compared to the actual blood glucose value. The latter is more stable than the two protocols, with an uncertainty of ±30 mg/dL in the glucose concentration level at a 90% confidence level (Figure 3C). However, the detection sensitivity was still unsatisfactory in the range of physiological glucose concentrations. Although the results of this program do not require advanced data processing involving extended wavelength ranges such as principal component analysis (PCA), subsequent improvements by adding additional array sets of QCLs may be required. To improve the selectivity and sensitivity of MIR spectroscopy, Kuhne et al. [48] employed surface-enhanced infrared absorption (SEIRA) spectroscopy, which exploits the unique molecular vibrational properties of the MIR region for the specific identification of chemical substances (Figure 3D). The subsequent combination of the PCA algorithm resulted in a good increase in the sensitivity and specificity of the sensor in pure aqueous fructose and glucose solutions of 10 g/L.

Usually, the measurement site of NIR will be selected in the fingers, earlobes, forearms, lips, oral mucosa, etc. But the measurements are less accurate on the fingers, and more ideal on the inside of the lips, where blood glucose levels are correlated with optical density [49,50]. In addition, glucose makes up only 0.07–0.1% of plasma, and other components of tissues or blood (including water, pigments, proteins, etc.) can affect the amount of light absorbed and interfere with the determination of blood glucose [51,52,53,54]. Therefore, the wavelength of the light source needs to be chosen in a range that is as highly specifically absorbed by glucose as possible. Although there are examples of NIR/MIR spectroscopic non-invasive glucose sensors that have been successfully used commercially, the sensitivity and selectivity of the sensors, the correlation between measurement results and actual values, and the subsequent algorithms used need to be further improved and modified.

### 3.2. Optical Polarimetry (OP)

Optical polarimetry (OP) is one of the earliest non-invasive blood glucose detection methods. Since glucose is an optically active substance and has stable optical rotation, when a polarized beam of light illuminates a solution containing glucose solutes, the presence of glucose causes a certain rotation of the polarized plane of the incident light. At this point, the polarization direction will form a deflection angle with the original incident direction, which is proportional to the amount of glucose [55,56,57,58,59]. After the polarized light has passed through the sample, the plane of the polarized light can also be measured with a polarimeter. When the polarization axis of the analyzer is matched to the rotation angle of the electric field, the photodetector will detect the maximum intensity of light. When the polarization axis is perpendicular to the rotation angle of the electric field, the light will not be detected by the photodetector [60,61]. Although the method is directly detected by visible light, not complicated to operate and easy to obtain results, the error accuracy of the method may not be satisfactory [62,63,64], mainly due to the following three reasons: (a) Affected by the scattering characteristics of the objects to be measured, polarized light is prone to lose its polarization, while human skin has a strong scattering property, and the optical activity of glucose is almost completely inhibited by the linear birefringence of dermis, so the eyes (especially the aqueous humor in front) are usually selected for measurement [55]. (b) When blood glucose is detected through aqueous humor of the eye, motion artifacts can cause changes in corneal birefringence. This time-varying birefringence is one of the most important interference source, which can significantly cause changes in the polarization state of detected light and thus confuse the optical characteristics of glucose [65]. (c) When the light beam passes through the skin and reaches the blood, the presence of biomolecules with optical rotation characteristics in the blood (such as vitamin C and albumin) could cause deviation that cannot be ignored in the deflection angle [56].

**Figure 4:**
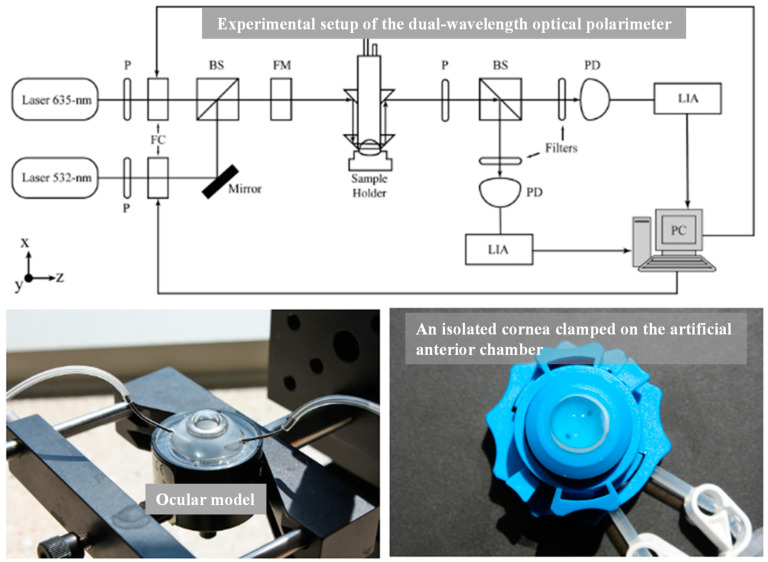
Optical polarimetry method: dual-wavelength polarimetric glucose sensing. Adapted with permission [67]. Copyright 2013, SPIE.

At present, there are basically two mechanisms to solve the interference problem of corneal birefringence in the polarization of blood glucose monitoring: a birefringence compensator and dual-wavelength polarizer [55,65,66]. Malik et al. proposed a dual-wavelength polarization glucose-sensing technique, in which the average standard error of prediction reached 125.4 mg dL^−1^ and 151.1 mg dL^−1^ in the range of 0–600 mg dL^−1^ glucose solution when the glucose concentration in the artificial anterior cornea was measured by polarization method at the wavelength of 635 nm and 532 nm, respectively. Combined with multiple linear regression (MLR) analysis, the mean error was reduced to 22.4 mg dL^−1^ and the linear correlation coefficient was 0.996 (Figure 4) [67]. However, this experiment only carried out in vitro detection, and there was no data from live experiments for reference, so the accuracy of the clinical measurement may not have been convincing. In addition, the complex experimental devices and procedures may not be conducive to home-based blood glucose monitoring.

Overall, although the OP method has shown its ability to detect glucose, its detection is subject to many limitations, in particular, the polarization determination of glucose in cloudy media is strongly influenced by scattering. This poses a significant challenge to the robustness and reproducibility of the sensor. In order to reduce interference, a large part of the research has focused on the measurement of water in the anterior chamber. A number of limitations have hindered the development of this sensor and there is still a long way to go before its practical possibilities for users are resolved.

### 3.3. Raman Spectroscopy

Raman scattering, similar to the Compton effect of X-ray scattering, is a special effect in the scattering phenomenon of light. When a laser of a certain frequency hits the surface of a sample, molecules in the material absorb some of the energy, vibrate in different ways and degrees, and scatter light of different frequencies. The variation in frequency depends on the nature of the scattered material. Different radicals vibrate in unique ways, generating scattered light at specific frequencies. The types of molecules that make up a substance could be identified by Raman spectroscopy [68].

**Figure 5:**
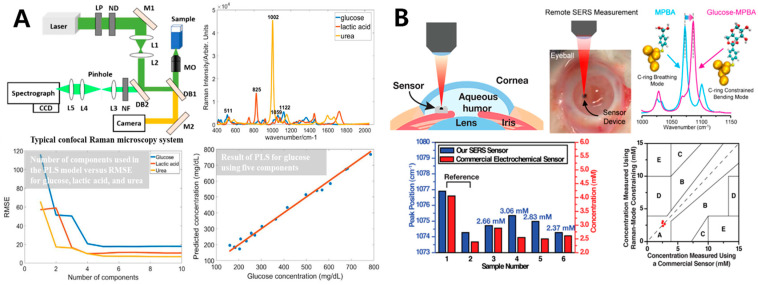
Raman spectroscopy methods. (**A**) multicomponent analysis using a confocal Raman microscope. Adapted with permission [68]. Copyright 2018, Optical Society of America. (**B**) Glucose sensing using surface-enhanced Raman-mode constraining. Adapted with permission [69]. Copyright 2018, American Chemical Society.

Based on the Raman scattering effect, the glucose concentration could be quantitatively analyzed. Compared with other optical methods, Raman spectroscopy detects the fundamental vibration of atomic groups, with less overlap and interference, and the identification results are more accurate. However, the indistinctive Raman scattering cross section of glucose may result in extremely weak Raman signal. Additionally, the Raman spectrum of glucose could be easily masked by the strong background noise of the surrounding environment [69]. In most cases, after spectral percutaneous measurements, multivariate stoichiometric modeling methods (e.g., partial least squares regression (PLSR), principal component regression (PCR), or support vector regression (SVR)) are used for feature extraction and cross-validation [70]. In order to weaken the interference of strong background signals generated by surrounding tissues, Shao et al. focused the laser on the blood in the blood vessels of the skin and calculated the blood glucose concentration with the hemoglobin concentration as the internal standard and the ratio of the height of 1125 cm^−1^ against 1549 cm^−1^. The results of a mouse experiment showed that the Raman intensity was roughly linear with the blood glucose concentration, a mean absolute error (MAE) in the validated data of 5.7% and an adjusted R-Square (Adj. R-Square) of 0.91 [71]. Tang et al. conducted multi-component analysis with confocal Raman microscope, and quantified physiological glucose concentration by partial least-squares regression method, with the predicted root mean square error (RMSE) of 17.81 mg dL^−1^ and the linear correlation coefficient of 93% (Figure 5A) [68]. In the latest research, Yang et al. proposed that glucose-MPBA (mercaptophenylboronic acid) bonding modifies two of MPBA’s Raman modes, resulting in a peak shift whose magnitude can be correlated to the ambient glucose concentration. Moreover, by tracking the glucose-induced shift in the surface-enhanced Raman-scattering (SERS) emission of MPBA, and using the SERS based on nano-structure to enlarge the weak Raman signal by 10^6^ to 10^8^ times, the rapid continuous glucose sensing within the physiological range of 0.1 mM to 30 mM was realized. In the experiment of intraocular blood glucose measurement in 6 isolated rabbit eyes, the detection error range is within 0.5 mM, which could be used for diabetic patients whose blood glucose concentration regularly changes around 10 mM (Figure 5B) [69].

Although these research results provide a lot of sustenance for the blood glucose monitoring by Raman spectroscopy, the chief obstacle confronted by a spectral glucose sensor in routine clinical application is still the inability to find an appropriate standard to analyze indefinite samples. Differences in skin tone, skin thickness, basal metabolic rate and hydration status frequently lead to non-linear variances in glucose concentration between dissimilar individuals and diverse parts of the body [70].

### 3.4. Fluorescence Method

Primarily, the decay of a molecule from a higher energy excited state back to the ground state through a relaxation process and emitting photons is called a radiation relaxation phenomenon. Radiation relaxation involves fluorescence and phosphorescence, and there are a large number of fluorescent groups in human blood, which can emit fluorescence when they are in an excited state [72]. Fluorescence sensors not only have the advantage of high sensitivity, but also the characteristic emission spectra of specific fluorophores ensure high specificity of the sensor. The quantum yield, photostability, absorption and wavelength of the fluorophore are all factors to be considered in a fluorescence sensor.

**Figure 6:**
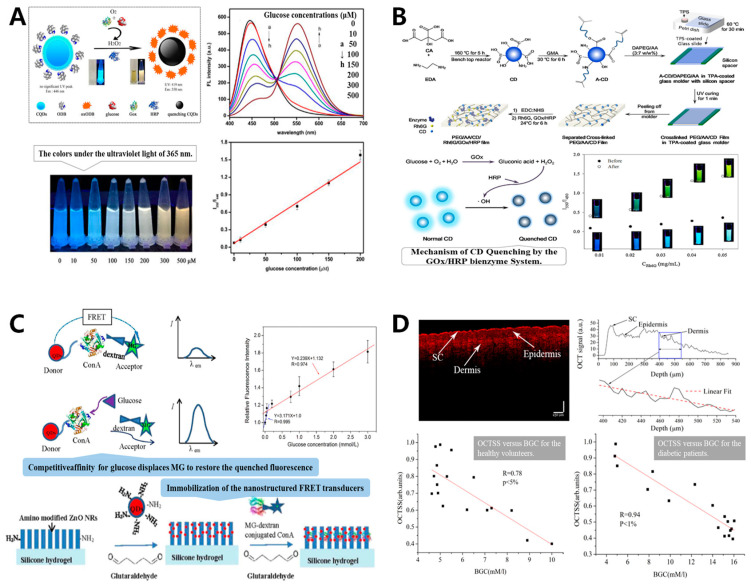
Fluorescence methods. (**A**) Colorimetric and ratiometric fluorescence dual-mode sensing of glucose. Adapted with permission [75]. Copyright 2019, MDPI. (**B**) Carbon-dot-based ratiometric fluorescence glucose biosensor. Adapted with permission [73]. Copyright 2019, Elsevier. (**C**) Nanostructured biosensor for detecting glucose in tear by applying fluorescence resonance energy transfer quenching mechanism. Adapted with permission [76]. Copyright 2017, Elsevier. Optical coherence tomography (OCT) method: (**D**) Non-invasive monitoring of blood glucose concentration in diabetic patients with optical coherence tomography. Adapted with permission [77]. Copyright 2017, Laser Physics Letters.

Some fluorescent materials, such as quantum dots (QDs) and carbon dots (CDs), are used as fluorescent probes for glucose detection. For example, H_2_O_2_ produced by enzymatic reaction between GOx and glucose can cause the fluorescence-quenching effect of QDs. The quenching degree represents the amount of glucose, which can be detected by colorimetric sensor on a macro level [73]. Zhai et al. develop a new fluorescence ratiometric sensor with CDs for glucose detection by using the theory that m-Dihydroxybenzene (mDHB) oxide can generate fluorescence and also quench the fluorescence of CDs. Although mDHB is not fluorescently excited, its oxides can produce fluorescence and quench the fluorescence of CDs. Glucose oxidation catalyzed by GOx generates H_2_O_2_, which is then quantitatively oxidized to mDHB by horseradish peroxidase (HRP), so that a quantitative relationship is constructed between the mDHB oxides and glucose. The generated mDHB oxides then cause a change in the fluorescence ratio between CDs and mDHB oxides, which is the basic principle of this fluorescence ratiometric glucose sensor. The results showed that glucose content had a good linear relationship with the fluorescence ratio of mDHB oxide and CDs, and the detection limit can reach 0.35 mM [74]. Subsequently, Zhai et al. constructed a fluorescence colorimetric sensor using the fluorescence-bursting effect of carbon quantum dots (CQDs) and o-diaminobenzene (ODB). ODB was quantitatively oxidized by the intermediate product H_2_O_2_ through a dual enzymatic effect of GOx and HRP. The ODB oxides (oxODB) generated were able to emit fluorescence (550 nm) and burst the fluorescence produced by CQDs (446 nm). The oxODB content varies with glucose concentration, which causes changes in the fluorescence ratio (I550/I446) and the absorbance ratio. Macroscopically, under sunlight and UV light, the color of the detection solution varies with the glucose concentration, which is the basic principle of the fluorescence colorimetric sensors. The results showed a good linear relationship between absorbance ratio and glucose concentration (10–500 µM), with a correlation coefficient R^2^ of 0.9944 and a LOD of 3.00 µM. In addition, the color of the detection solution showed a gradient change under sunlight (Figure 6A) [75]. Cho et al. designed a ratiometric fluorescence glucose biosensor based on CDs and rhodamine 6G (Rh6G). A ratiometric fluorescence color change was achieved by the bienzymatic reaction of GOx and HRP with glucose through fluorescence quenching. Based on this principle, the team designed a stable solid biosensor membrane with a linear range of 0.5–500 μM, a LOD of 0.08 μM, and the ratiometric fluorescence color gradually turned from blue to green as the glucose concentration increased (Figure 6B) [73]. Although the ratiometric biosensors improve the ability to detect glucose with the naked eye, it is unfortunate that accurate quantitative analysis cannot be achieved. In addition, Chen et al. used the fluorescence resonance energy transfer (FRET) quenching mechanism to develop a nanostructured biosensor for the detection of glucose in tears. The relative fluorescence intensity increased linearly with the glucose concentration from 0.03 mM to 3 mM, and the linear correlation coefficient was 0.974 (Figure 6C) [76].

Under laboratory conditions, these fluorescence sensors have shown better sensitivity and detection range, so in vitro testing projects are more common. However, the defects of fluorescence sensors are evident, as they usually measure isolated body fluids, such as tears and sweat, which are poorly correlated with blood glucose, and also suffer from low signal-to-noise ratio, detection hysteresis and inability to quantify.

### 3.5. Optical Coherence Tomography (OCT)

Optical coherence tomography (OCT) is a new kind of high-resolution imaging technology. OCT provides depth-oriented tomography capabilities based on low-coherence interference. The contrast of OCT signals results from spatial variations in optical reflection (scattering) properties within biological tissues or materials. It can observe the internal microstructure of biological tissue 1~10 mm deep without destroying the integrity of biological tissue and its spatial resolution can reach 10~15 microns [77]. OCT has the distinct advantage of being able to measure and analyze a specific skin fault, not only reducing interference caused by other factors, but also having high resolution, high signal to noise ratio, high penetration depth and not being affected by blood pressure, heart rate and hematocrit [5]. However, sensitivity to skin temperature change and movement [5], low accuracy of measurement, histone fluid glucose hysteresis, universality and repeatability of calibration algorithms are all key factors restricting its development [78].

The distinction of glucose concentration will lead to a different dermal tissue scattering coefficient, which will result in the OCT signal slope (OCTSS) change [77]. OCT technology can measure the variable of OCTSS directly from dermis when performing non-invasive blood glucose detection. Esenaliev et al. found that the OCTSS declined linearly with the increase of blood glucose concentration [79]. Lan et al. observed that the correlation coefficient R between blood glucose concentration and OCTSS in diabetic was 0.91, while that of healthy volunteers was only 0.78, suggesting that OCT technology’s detection ability and accuracy in diabetics may be better than that of healthy people (Figure 6D) [77]. However, Pretto et al. used osmotic shock method for OCT analysis of isolated samples [80]. Their study showed that the resolution was greatly affected by the presence of large multiple scattering components. In addition, under the hyperglicemic condition, the attenuation coefficient may be affected by erythrocyte deformation, so it may not be suitable for direct application in human body.

In a word, the objective of the optical method is to estimate blood glucose levels indirectly through the specific response of various light waves to glucose concentration in the body, rather than by direct measurement. Although the detection of human blood glucose by optometry could achieve non-invasive conditions, the essential reason why it cannot be applied in clinical and commercial practice is that the measured value is not highly correlated with the actual blood glucose value, and the linear range is narrow, which requires subsequent algorithm correction. However, there is a large difference between individuals, so the current fitting method based on experimental samples cannot be fully applicable to all individuals. In addition, complex detection methods, harsh detection sites, cumbersome detection process, high requirements for detection equipment, and large interference of background signals also limit the prospect of its use as a family commercial glucose meter.

## 4. Non-Invasive Blood Glucose Monitoring Technology—Microwave Method

In the past few decades, with the rapid development and widespread application of wireless technology, the comfort, portability and integration of various electronic products have been greatly improved, including more affordable [81]. In medical diagnosis is no exception, the interaction between electromagnetic waves and biological tissues has attracted widespread attention. Microwaves, which are electromagnetic waves with wavelengths between 1 mm and 1 m, corresponding to frequencies between 300 GHz and 300 MHz [82], can easily penetrate biological tissues of millimeter thickness, especially in the low-frequency range, offering advantages not found in some optical detection methods. This is because microwave radiation has a lower energy per photon and is less scattered in the atmosphere, suggesting that they can penetrate deeper into the tissues and obtain more realistic blood glucose data [5]. In general, the design of microwave sensors is based on the fact that reflection, transmission, and absorption of microwaves are closely related to the dielectric properties of tissues, and the dielectric constant varies with glucose fluctuations [5,83,84]. However, exactly where in the tissue layer the changes occur and the mechanism by which the dielectric properties change is unclear [85]. The dielectric properties of biological tissues have been widely reported in the literature [86]. The difference of dielectric properties between normal and abnormal tissues is the core of microwave diagnosis. In recent years, in the non-invasive continuous blood glucose monitoring, the correlation between glucose concentration in blood or other biofluids and its dielectric properties has attracted the attention of many researchers, and has led to further research on microwave sensors [87].

**Figure 7:**
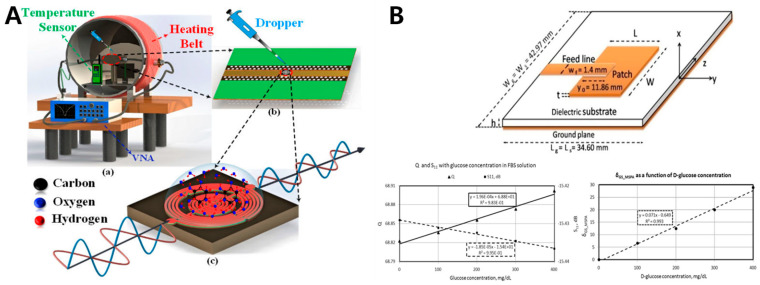
Microwave methods. (**A**) High-sensitivity, quantified, linear and mediator-free resonator-based microwave biosensor for glucose detection. (a) Microwave-based glucose biosensor, (b) micro-fabricated biochip mounted printed circuit board (PCB), and (c) sensing mechanism of microwave biosensor. Adapted with permission [91]. Copyright 2020, MDPI. (**B**) Demonstration of microstrip sensor for the feasibility study of non-invasive blood-glucose sensing. Adapted with permission [92]. Copyright 2020, Springer.

It has been shown that the sensitivity is positively correlated with both the fill factor and the load quality factor (Q). In general, the high Q value is more favorable to the sensitivity of the sensor due to its ability to enhance the local electric field strength [88,89]. However, at the same time, the Q value also depends on the dielectric loss, transmission loss and parasitic losses of the material [90]. Advanced micro-fabrication techniques can be used to improve the filler factor. Currently, Kumar et al. [91] employed an intertwined air-bridge-type asymmetrical differential spiral inductor, achieving high inductance values by adjusting the Q and introducing a centralized inter-digital capacitor to produce an enhanced electric field (Figure 7A). The microwave biosensor not only possessed a low resonance frequency (1.50 GHz) and high penetration depth (34.46 mm in DI water), but also exhibited high sensitivity (117.5 MHz/mgmL^−1^), linear response (r^2^ = 0.9987) and maximum reproducibility (0.78%). Satish et al. [92] designed a Microstrip Patch Antenna Sensor that resonance frequency at 2.4 GHZ. Without ionization and direct contact with biological tissue, the electromagnetic coupling of microwave signal could penetrate subcutaneous biological tissue to the blood layer. When blood glucose levels fluctuate, the variation in blood and its underlying tissues dielectric properties would affect the sensor’s reflection-based parameters. They defined a novel multi-parameter sensitivity factor to more comprehensively evaluate the response of the sensor to changes in glucose concentration. The sensitivity factor was jointly defined by four parameters, namely, input reflection coefficient (S_11_), resonance frequency (f_r_), half-power bandwidth (BW) and quality factor (Q). The results showed a good correlation (R^2^ = 0.993) between the reflection performance and the D-glucose concentration (0–400 mg/dL) (Figure 7B). Omer et al. proposed that low-cost mm-wave radar in the range of 50–67 GHz has a high sensitivity to detect glucose concentration, which is mainly used to identify different blood glucose concentrations through correlation with the reflected mm-wave readings. In synthetic blood samples with glucose concentration of 0.5–3.5 mg/mL, the unique multi-channel original radar signals of different glucose samples were recorded and analyzed by signal processing algorithms, so that the radar signals could successfully distinguish the unique dielectric properties of each blood sample [93].

However, finding the relationship between blood glucose levels and the effective permittivity remains a central challenge for microwave sensors [5,81,82]. In other words, current research focuses on how to improve the selectivity and sensitivity of microwave sensors. In terms of selectivity, the dielectric properties of tissues are simply an average value, and its variations may be caused by a combination of changes in multiple components of the tissue. How to attribute measured changes in dielectric properties to blood glucose may require subsequent ongoing exploration. Multi-sensor integration, big data computing and machine-learning techniques may weaken the influence of internal and external factors to some extent, which is worth thinking about [89,91,94]. In terms of sensitivity, changes in human blood glucose concentrations produce only slight changes in dielectric properties, which requires a highly sensitive sensor. This also means that it is necessary not only to have a high-precision, high-stability, multi-frequency resonator to attenuate the effects of noise, but also to subsequently employ appropriate signal-processing technology to extract low amplitude variations in the case of large amplitude variations [83,88,90,95,96].

In general, microwave sensors are favored by many researchers in the field of non-invasive blood glucose and have a broad development prospect due to their high penetration depth, non-ionization, low cost and portability. However, their poor sensitivity and selectivity are still the core factors limiting their development, and further efforts should be made towards these two aspects in the future.

## 5. Non-Invasive Blood Glucose Monitoring Technology—Electrochemical Methods

### 5.1. Reverse Iontophoresis (RI) Technology

ISF is the extracellular fluid that surrounds tissue cells. It is composed of many important biomarkers and has similar medical diagnostic potential to blood. Small molecular biomarkers are exchanged between blood and ISF through diffusion. Therefore, the correlation between ISF and blood can be used to obtain the health information of patients indirectly. Transdermal reverse iontophoresis (RI) is a needle-free technique, which can extract biomolecules and drugs through intact skin to achieve the purpose of blood glucose detection.

An example of the great use of RI is GlucoWatch [97,98], which consists roughly of hydrogel disks, iontophoresis electrodes and biosensors. The hydrogel disk contains GOx, which reacts with glucose extracted by RI. When the device is worn like a watch, it produces a current (300 μA) which affected the subdermal molecules. Under the influence of the electric field, positive ions move towards the cathode terminal, negative ions towards the anode, and neutral glucose molecules move towards the cathode owing to the influence of electroosmosis [99]. The sensor can recognize the number of electrons transferred in proportion to the glucose molecules extracted. The current signal is converted into corresponding blood glucose level by a data conversion algorithm. However, the device has many limitations, such as the need to use a standard glucometer for calibration, a long warm-up time, the need to change the gaskets every 12 h, the device cannot be used during sweating, and prolonged electric current can cause skin damage to users, etc. Sales of the device stopped in 2007 [100,101]. However, the GlucoWatch was the first FDA-approved non-invasive blood glucose monitoring product to be marketed using RI technology. It served as a very classic RI prototype and had a very profound impact on many of the RI sensors that followed.

**Figure 8:**
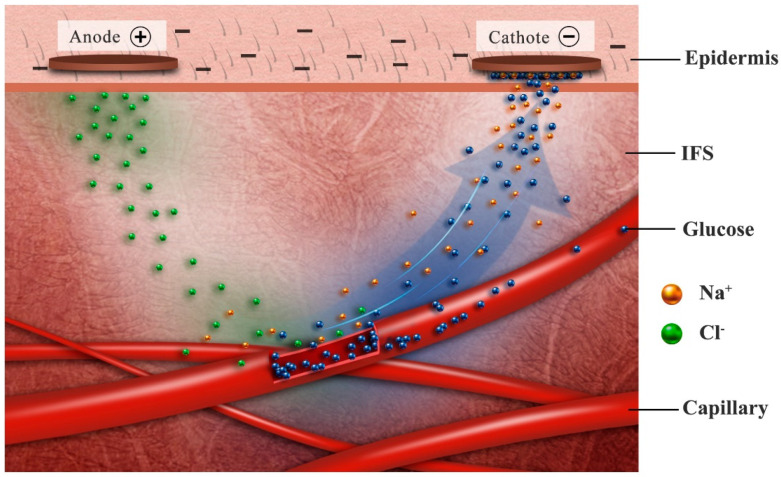
Schematic diagram of reverse iontophoresis (RI).

As shown in Figure 8, RI involves two mechanisms: (i) electromigration, the direct interaction between charged ions and applied electric field, and (ii) electroosmosis, a connective solvent flow from anode to cathode direction. For the charged moieties, electromigration is the main transport mechanism. The total amount of charge transferred depends on the strength and duration of the electric field. For the uncharged moieties, electroosmosis driven by voltage is the main extraction mechanism. As the main charge carrier, the electromigration of ${\mathrm{Na}}^{+}$ forms an ion current from anode to cathode. The ions in ISF are mainly composed of ${\mathrm{Na}}^{+}$ and ${\mathrm{Cl}}^{-}$, while under normal physiological conditions, the epidermis is negatively charged, so the skin will promote the transport of ${\mathrm{Na}}^{+}$, resulting in the electromigration number of ${\mathrm{Na}}^{+}$ is greater than ${\mathrm{Cl}}^{-}$. Therefore, NaCl on the anode decreased and NaCl on the cathode side increased during RI. This electrochemical gradient promotes the osmotic flow of water from the anode to the cathode, and the neutral molecules in ISF also move towards the cathode along with the direction of the percolation. Therefore, cations and neutral substances can be extracted from cathode and anions can be extracted from anode by RI [100,101,102,103].

However, there are various factors that may affect the extraction effect of RI. In terms of extraction of glucose molecules, the main factors are as follows:

(1) Skin thickness

Ordinarily, the thicker the skin, the lower the permeability, and the worse the extraction effect. Skin thickness varies greatly among individuals, depending on races, age, gender, skin condition and disease state etc.

(2) Current intensity

Generally, RI adopts direct current and the relationship between current intensity and extraction amount is nearly linear. The increase of current intensity can increase the ion migration, but with the continuous increase of current intensity, the transfer rate tends to be stable, indicating the saturation state has been reached. At this time, a further increase of current intensity makes no significant contribution to the ion migration. In addition, the skin will form microscopic pores at high current density, and the electroporation may open the water-containing channel to increase the penetration of the skin, so as to promote the effect of RI [104]. This method of combining electroporation with RI may achieve a better extraction effect in a shorter extraction time and a smaller current. Zhao et al. [104] suggested that electroporation for 5 min followed by RI for 12 min yielded better results, indicating that the synergistic effect of electroporation and RI could promote skin penetration.

(3) Constant current or pulsed current

If direct current is applied to the skin continuously, it may cause tingling and erythema on the epidermis. Pulsed current can effectively reduce this side effect in RI. The pulse waveform gives the skin time to depolarize and come back to its original state before opening the next pulse, which significantly reduces irritation to the skin so that the patient can tolerate higher levels of current.

(4) Duration of current

When the current intensity is constant, glucose extraction is proportional to the duration of the current applied. The skin can return to normal within a period of time. But irreversible damage can occur when skin is exposed to high levels of current for a long time. Normally, the current intensity should be between 0.1 to 0.3 mA, and the duration of each time should not exceed 20 min.

(5) Electrode materials

The size, shape and location of electrodes are also crucial factors for RI. Current density is closely related to the electrode size. Electrodes come in a variety of shapes, from thin disks (like GlucoWatch) to snakes to ridges or otherwise. It is not only required that the electrode can be closely attached to the human skin, but also maintain an appropriate micro-distance from the skin surface. Hence, flexible materials and designs are generally selected for the electrode. Aluminum, platinum and zinc electrodes were used in the initial study. But they can cause water electrolysis, which causes pH changes and produces gases that accumulate on skin surfaces and electrodes, interfering with current distribution. To avoid electrochemical reaction of water on the electrodes, materials with low redox potential are generally used. For example, Ag/AgCl electrode has excellent biocompatibility, reversibility and resistance to pH shifts, so it is a appropriate candidate for the RI electrode materials.

At present, nano-gold electrode is also favored by many researchers. Its advantages are as follows: (a) nano-gold electrode has excellent electrochemical performance, such as better current density, more obvious redox characteristic peak, faster current response time and higher sensitivity than a conventional gold electrode; (b) the Young’s modulus and hardness of the nano-gold electrode are much lesser than those of the conventional gold electrode. This mechanical property of high elasticity and low hardness enables it to fit the human skin more tightly without easily falling off or breaking in the process of exercise, and it feels more comfortable; (c) the nano-gold electrode has excellent biocompatibility and surface effects. More surface active sites and higher surface adsorption capacity can substantially improve the catalytic activity of curing enzyme and increase the response current [105,106,107].

### 5.2. Non-Invasive Biofluid-Based (Saliva, Tears, Sweat and Interstitial Fluids (ISF)) Glucose Monitoring Devices Comparison

Table 1 shows the advantages and disadvantages of different types of glucose sensor and the current major research status owing to the differences in the glucose content and extraction methods of biofluids in different parts of the body. 

#### 5.2.1. Saliva-Based and Breath Acetone Glucose Monitoring

**Figure 9:**
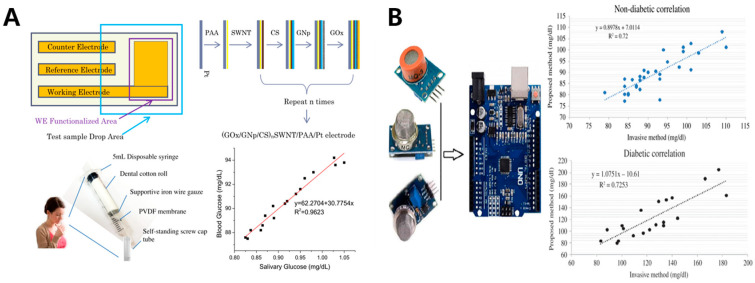
Saliva-based and breath acetone glucose monitoring. (**A**) Non-invasive glucose monitoring using saliva nano-biosensor. Adapted with permission [108]. Copyright 2015, Elsevier. (**B**) Non-invasive measurement of blood glucose by breath analysis. Adapted with permission [129]. Copyright 2020, John Wiley and Sons.

A saliva non-invasive glucose sensor is usually a mouthguard-type, which is made of biocompatible and non-toxic materials [18]. As is known, saliva is a clear and sticky biofluid secreted by salivary glands into the mouth. It contains a variety of biomarkers, such as glucose, lactic acid, phosphate, enzymes (such as amylase), hormones (such as cortisol, steroids), antibodies and so on [112]. Saliva is easier to extract and collect than other biofluids, and its richness and convenience facilitate the study of saliva biomarkers, which is a major advantage of saliva-based glucose monitoring. Zhang et al. designed a disposable saliva nano-biosensor that can be used to detect saliva in vitro [108]. Through a layer-by-layer (LBL) assembly technique, single-walled carbon nanotubes (SWNT), multilayers of chitosan (CS), gold nanoparticles (GNp) and glucose oxidase (GOx) were successively assembled as working electrodes. After filtering the unstimulated saliva samples, the results showed that a reliable linear detection range was between 0.017–1.11 mM with sensitivity of 26.6 ${\mu \mathrm{AmM}}^{-1}{\mathrm{cm}}^{-2}$ and correlation coefficient of 0.995 (Figure 9A) [108]. In clinical trials, they found a good correlation between glucose levels in saliva and blood before and two hours after glucose intake. The correlation between fasting glucose and salivary glucose in healthy subjects was approximately 0.96.

Although saliva is a reasonably attractive candidate for non-invasive blood glucose monitoring, significant obstacles remain in the practical detection process. Above all, it will seriously interfere with the extraction, detection and sensing of target analytes that the decomposition of food residues could lead to the production of a large number of secreted proteins and active chemicals and other interfering impurities during continuous monitoring [18]. Moreover, Naseri et al. compared the saliva test reports of 1432 diabetic patients and 900 healthy control group, and the results showed that the correlation coefficient between saliva and blood glucose of patients was not satisfactory (*r* = 0.765), which was lower than that of healthy people (*r* = 0.646) [113]. Therefore, saliva-based sensors should be required to maintain high specificity in this complex and dynamic chemical environment. More seriously, there are temperature and humidity conditions suitable for the growth and reproduction of bacteria inside the mouth, which may cause them to accumulate rapidly on the surface of the sensor forming biofilms, affecting the sensitivity [114]. Thus, in the subsequent design of wearable saliva-based sensor, in addition to the general electrochemical ability to be considered, it is necessary to find appropriate solutions in response to the mechanical stress caused by oral movement and teeth, antimicrobial coating and high-specificity recognition. Furthermore, there were differences in the composition and concentration of unstimulated and stimulated saliva, as well as saliva produced by different methods [115].

Among the methods of glucose monitoring of oral secretions, in addition to this common direct detection of saliva, there is another option—a non-invasive glucose sensor based on breath acetone—that is also worth considering. In healthy individuals, the process by which fatty acids are broken down into ketones, a high-energy compound, is known as ketogenesis, and the rate of ketogenesis is regulated by insulin and glucagon. In short, insulin inhibits ketogenesis and glucagon stimulates ketogenesis. In diabetic patients, insulin deficiency leads to an increase in acetone content, which is the pathogenesis of diabetic ketoacidosis (DKA) [36,116,117]. Acetone can leave the body through respiration. In general, the amount of acetone in exhaled air ranges from 0.2~1.8 ppm in healthy people to 1.25~2.5 ppm in diabetics, and even up to 25 ppm in type-1 diabetes [118]. The breath acetone-based non-invasive glucose sensor is a new detection method to indirectly infer the blood glucose concentration by detecting the acetone content in the exhaled air of the human body.

However, the correlation between breath acetone and blood glucose concentrations reported in the literature is very uncertain (positive [119,120], negative [121], no correlation [117,122,123]), especially for single measurements. This is because there are many factors that can influence acetone production, and varying experimental conditions will alter the correlation. For example, insulin injections, food intake, strenuous exercise, alcohol intake, individual physiological differences, type of diabetes, and other types of disease can all have an impact on the rate of acetone metabolism [36,124,125,126]. These factors make the relationship between breath acetone and blood glucose levels not simple and linear. In fact, this is the core problem of breath acetone-based non-invasive glucose sensor [125,126,127,128]. Secondly, acetone is a product of fat metabolism, which is not directly produced by glucose, so there may be a time lag in the mapping change of blood glucose and respiratory acetone. The exact lag time also varies from person to person and does not have a very precise range [116,117,129]. There are generally two better directions to solve the problem: one is to perform multi-line sensing analysis in combination with other breath analytes to reduce the inaccuracy of a single measurement and improve the predictive ability. For example, Mansouri et al. [129] combined three breath analyte sensors, alcohol, acetone and propane, and by multiple linear regression (*r* = 0.97 for healthy subjects and *r* = 0.35 for diabetics), obtained an analytical model with a relative error of 3.93% for blood glucose in non-diabetic subjects, but an error of 13.42% for diabetic subjects (Figure 9B). The other is to simulate a more realistic model by expanding the sample extraction, coupled with the big data computation of the neural network model.

#### 5.2.2. Tear-Based Glucose Monitoring

**Figure 10:**
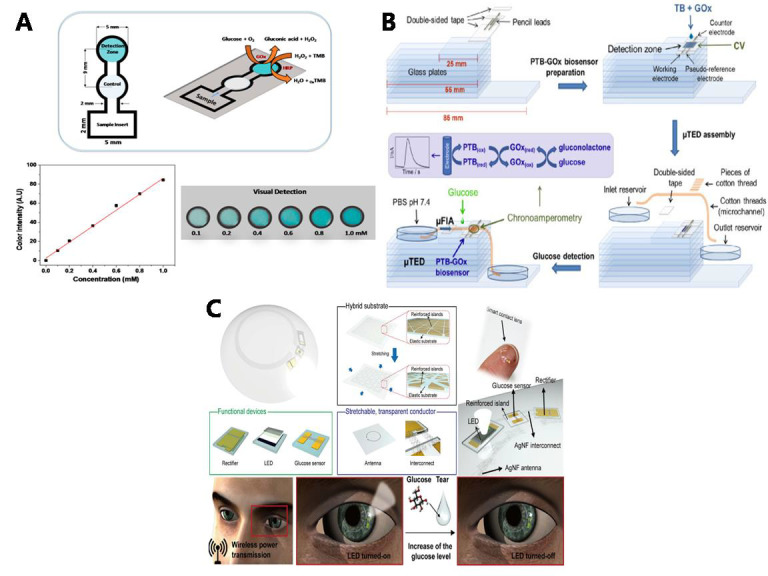
Tear-based glucose monitoring. (**A**) Paper-based colorimetric biosensor for tear glucose measurements. Adapted with permission [131]. Copyright 2017, Micromachines. (**B**) Tear glucose detection combining microfluidic thread based device, amperometric biosensor and microflow injection analysis. Adapted with permission [109]. Copyright 2017, Elsevier. (**C**) Soft, smart contact lenses with integrations of wireless circuits, glucose sensors, and displays. Adapted with permission [137]. Copyright 2018, Science advances.

Tears are also a biomarker-rich liquid, which contains a lot of salt, protein, enzymes and glucose. Some useful information about the condition of the eyes and parts of the body could be obtained by analyzing their chemical composition. The main advantage of the tear-based non-invasive glucose sensor is that there are fewer interference impurities in tears and the glucose in tears is positively correlated with blood glucose [130]. Ellen et al. designed a chitosan modified paper-based colorimetric biosensor, which can obtain glucose concentration by detecting in vitro tears and colorimetric analysis with standard samples (Figure 10A) [131]. The achieved analytical sensitivity and LOD were 84 AU mM^−1^ and 50 µM, respectively. The paper-based colorimetric biosensor exhibited a linear relationship in the glucose concentration range between 0.1 and 1.0 mM. Augustini et al. designed an electroanalytical microfluidic device based on cotton threads, which detected tear samples for microflow injection analysis. The pencil leads coated with an electropolymerized film of poly (toluidine blue O) and glucose oxidase (PTB-GOx) as the working electrode of the sensor. The measurement results of tear glucose showed high stability (RSD = 2.54%), and a wide linear range between 0.075 and 7.5 mM, the linear coefficient was 0.961, and the detection limit was 22.2 μM (Figure 10B) [109].

Initial tear-based glucose sensors were typically designed as strips and attached to a flexible stretchable substrate, but they have been phased out because they are difficult to attach to the pupil [114]. Currently, many research teams are focusing on contact lens-type glucose sensors for user portability and sustainability of glucose monitoring. Not only can it realize non-invasive continuous detection, but also can transmit real-time data to the user’s mobile terminal through wireless transmission, which is convenient for users and doctors to observe and regulate blood glucose level, and has a broad market prospect. However, the contact lens-type tear glucose sensor still faces many problems and has not yet reached the standard to enter the market [132]. First of all, the transmittance of a contact lens will decrease owing to the sensor and transmission circuit, which requires increasing transmittance in material selection and circuit design. Secondly, for the convenience of users, the sensor generally adopts a wireless power supply and wireless transmission, which puts forward high requirements for the energy supply of signal transmission. If the sensor generates heat during use, it will cause irritation and discomfort to the eyes. In the future, bio-fuel cells may be combined with sensors to solve the problem of energy supply for signal transmission [133]. In addition, Jeffrey et al. detected that the average lag time between tear glucose and blood glucose reached 13 min, and the correlation coefficient $R^{2}$ was only 0.7544 [134], so the measurement accuracy could not meet the clinical requirements. In the early stage, the linear interval of the contact lens-type glucose sensors developed by Chu et al. was 0.03–5.0 mm and the correlation coefficient was 0.999, but the delay was about 10 min compared with the blood glucose value [135]. Recently, Park et al. designed a flexible smart contact lens that used a silver nanofiber spinning network as a transparent stretchy circuit and integrated a graphene glucose sensor and wireless display on an elastic composite substrate. Although the lens is 93% transparent in visible light, has a response time of 1.3 s, a detection limit of 12.57 microns, and is optimized for heat, unfortunately the device does not provide a quantitative measure of tear glucose concentration (Figure 10C) [136,137].

#### 5.2.3. Sweat-Based Glucose Monitoring

**Figure 11:**
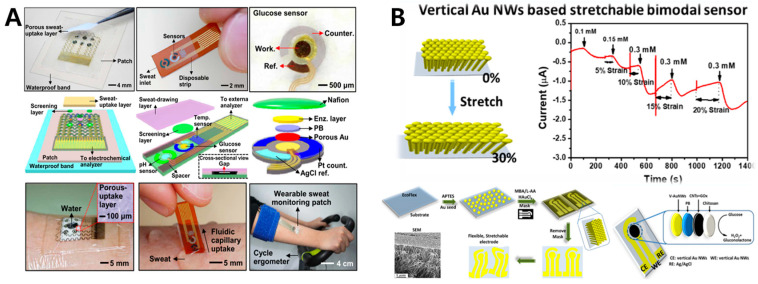
Sweat-based glucose monitoring. (**A**) Wearable/disposable sweat-based glucose monitoring device with multistage transdermal drug delivery module. Adapted with permission [138]. Copyright 2017, Science advances. (**B**) Enokitake mushroom-like standing gold nanowires toward wearable noninvasive bimodal glucose and strain sensing. Adapted with permission [110]. Copyright 2019, American Chemical Society.

A non-invasive sweat-based glucose sensor is also one of the most studied types of sensor in recent years. Sweat is a biological fluid secreted by sweat glands that contains metabolites, electrolytes, trace elements, and biological macromolecules. The presence of blood-related biomarkers in sweat makes it a hot medium for non-invasive monitoring. The design of patch or band-type makes it possible for it to be wearable and continuous, and the combination of other drug-delivery modules could achieve the purpose of continuous monitoring blood glucose and intelligent closed-loop treatment. Lee et al. designed a wearable sweat-based glucose monitoring device with multistage transdermal drug delivery module (Figure 11A) [138]. The sensor-monitoring part of the device integrated glucose sensor, PH sensor, temperature sensor and humidity sensor, which greatly improved the sensor precision. In addition, the glucose content in sweat was detected and fed back to the multi-stage heating and drug-delivery module, then hypoglycemic drugs were released in a timely way and accurately through transdermal therapeutic system, realized non-invasive closed-loop blood glucose management. This transdermal delivery not only could eliminate the pain associated with the injection, but also bypassed the digestive system, resulting in lower doses than oral administration and no gastrointestinal side effects. However, this sweat-based glucose sensing still faces many challenges, such as difficulty in sweat collection, activity variation of GOx owing to lactic acid secretion and changes in ambient temperature, and delamination of the enzyme when exposed to mechanical friction and skin deformation. Zhai et al. designed a wearable non-invasive glucose sensor using enokitake mushroom-like standing gold nanowires. Considering the influence of different tensile strain on the detection sensitivity, when the strain were 0, 10%, 20% and 30%, the detection sensitivity would reach 25.45, 19.45, 11.79 and 4.55 $\mu A\cdot {\mathrm{mM}}^{-1}\cdot {\mathrm{cm}}^{-2}$ respectively. As the strain increased, the sensitivity decreased and the detection limit reached 10 μM (Figure 11B) [110].

The advantage of a non-invasive sweat-based glucose sensor is that the sweat can be easily sampled, and most of the devices are highly integrated and wearable, capable of continuous measurement and very comfortable for the human body. However, in general sweat can only be detected and analyzed when it reaches the surface of the skin, so how to collect sweat on the spot and in moderation is a unique limitation. At present, most schemes extract and collect sweat by means of long strenuous exercise, heating, pressure and ionization energy stimulation, but all have their disadvantages (for example, diabetics are not suitable to sweat through long period of exercise; heating and ionization stimulation will bring pain and discomfort). The relatively low sensitivity due to the less glucose content in sweat and the certain hysteresis relative to the blood glucose concentration are also the main aspect restricting its application [139,140,141].

#### 5.2.4. ISF-Based Glucose Monitoring

**Figure 12:**
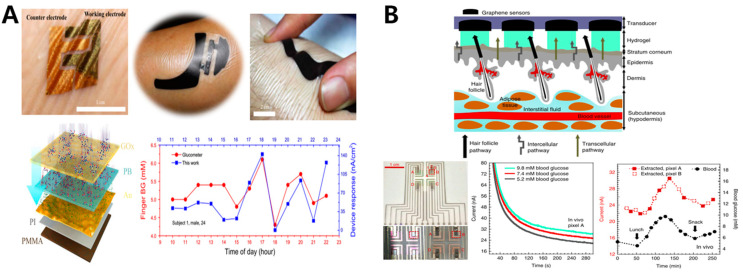
ISF-based glucose monitoring. (**A**) Skin-like biosensor system via electrochemical channels for noninvasive blood glucose monitoring. Adapted with permission [111]. Copyright 2017, Science advances. (**B**) Non-invasive, transdermal, path-selective and specific glucose monitoring via a graphene-based platform. Adapted with permission [142]. Copyright 2018, Nature Nanotechnology.

The ISF glucose sensor was used to extract glucose by transdermal RI. Its advantages include high glucose concentration in ISF, good linear range and high correlation coefficient with blood glucose, so its accuracy is much better than the other three methods. However, the problem we still face is that, on the one hand, a higher current is conducive to improving the detection limit and sensitivity of the sensor; on the other hand, the higher current will result in higher irritation to the skin, which may cause symptoms such as redness, swelling and tingling, and reduce the comfort level of users. Moreover, since the material exchange between blood and ISF exists for a certain period of time, the measured results often lag behind, and may not reflect the real-time value of blood glucose. Chen et al. designed a kind of skin-like biosensor system, which integrates an ultrathin (~3 mm) skin-like biosensor with paper battery–powered electrochemical twin channels. Its outstanding advantage was the fabrication and use of nanostructured gold films as a flexible electrode, which not only had better electrochemical characteristics than an original gold electrode, but also could fit tightly to the human skin. In in vivo human clinical trials, the high sensitivity (130.4 mA mM^−1^) and correlation coefficient (>0.9) reached excellent standards (Figure 12A) [111]. Lipani et al. designed a graphene electrode glucose sensor, a path-selective, non-invasive, transdermal glucose monitoring system, using follicular pathways in the skin as the privileged percutaneous extraction channel for electroosmotic extraction from ISF, and quantified the glucose value through the platform of the pixels of the array on the flexible substrate, which showed a good correlation with blood glucose (Figure 12B) [142]. Furthermore, considering if a constant current for a long time in the skin could cause cutaneous polarization and stimulation which could lead to lower permeability of the skin and damage such as red sparkling, the team used electrodes switching technology, the positive and negative electrodes of RI were exchanged a time in each cycle of electroosmosis, that greatly retarded the stimulation to the skin caused by RI technology.

**Figure 13:**
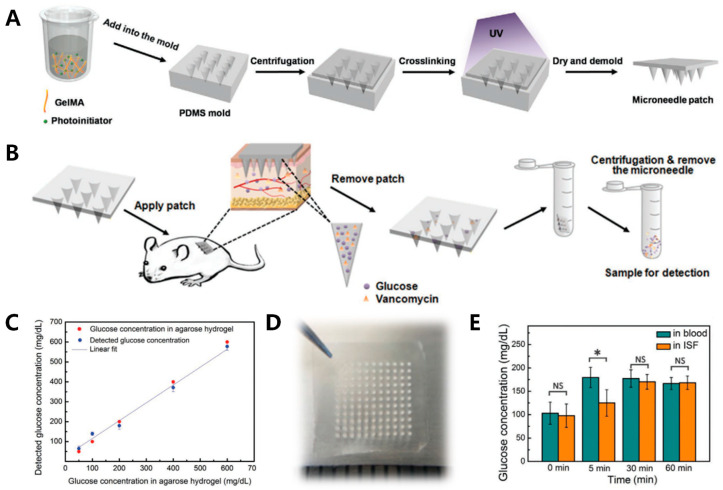
Gelatin methacryloyl microneedle patches for minimally invasive extraction of skin interstitial fluid. (**A**) Preparation process of the GelMA MN patch. (**B**) Schematic diagram of the extraction process in a rat model. (**C**) The detected glucose concentration (blue dots) was fitted as a line (blue line), R^2^ = 0.981. (**D**) A picture of the GelMA MN patch containing an 11 × 11 array of MNs over a 1 cm × 1 cm area. (**E**) Detected glucose concentrations in ISF compared to blood glucose concentrations. * *p* < 0.05, NS means not significant. Adapted with permission [147]. Copyright 2020, Small.

It is also a promising topic to improve the extraction rate of ISF by using microneedle array technology to break through the limitation of the stratum corneum. The microneedles (MNs) form temporary micron-size pores in the skin that are used as channels for molecular delivery, allowing them to enter or escape through the skin [143]. The length of the microneedle is generally between dozens of microns and a few millimeters, which ensures that it can puncture the cuticle, achieving the effect of transdermal drug delivery or RI without stimulating the neural network in the dermis. It is a painless and minimally invasive technique. Furthermore, although the microneedle patch leaves tiny puncture holes after use, this is only temporary. After the microneedle is removed or dissolved, the skin returns to its original condition, leaving no wounds that may cause skin infections [144,145,146]. Zhu et al. prepared dissolving gelatin methacryloyl (GelMA) microneedle patches. With its high swelling ratio and good biocompatibility, ISF was extracted into the hydrogel MN patches, and the glucose content in ISF in vivo was quantitatively and efficiently detected. In in vitro experiments using agarose hydrogel simulation, the detected glucose concentration was linearly correlated (R^2^ = 0.981) with the actual concentration of 50–600 mg dL^−1^. In in vivo experiments in rats, the microneedle patch collected about 2.5 mg ISF in 10 min and quantified glucose concentration. After removing the patch for 20 min, the skin recovered (Figure 13) [147].

## 6. Conclusions

This paper reviewed the research progress of non-invasive glucose monitoring technology in recent years. The new-type non-invasive blood glucose detection method could provide continuous (24/7) real-time blood glucose monitoring that solves the limitation of the traditional invasive blood glucose meter that requires repeated fingertip blood, which allows diabetics to monitor and manage their own blood glucose level conveniently, and has a broad market application prospect. Currently, non-invasive blood glucose monitoring methods are generally divided into three categories: optical methods, microwave methods and electrochemical methods. In general, the advantages of optical and microwave methods lie in their highly non-invasive nature and continuous monitoring without stimulating discomfort to the human body. However, as far as the research status goes, the measured value may be not highly correlated with the actual blood glucose value and the linear range is narrow, so subsequent algorithm correction should be required. For the sample part, individual differences (including age, skin color, skin condition, etc.) will cause large errors to the measurement results, resulting in the consistency, stability and reliability of the instrument not being strongly proved. As for the detection part, there are still some problems such as complicated detection means, harsh detection parts, a tedious detection process, high requirements for detection equipment, and large interference of background signals. The above three limitations may limit its application prospect as a family commercial glucose meter. Electrochemical methods mainly make use of the correlation between some biofluids (such as saliva, tears, sweat, and ISF) and blood glucose concentration, and indirectly obtain the blood glucose value by measuring the glucose content in these body fluids. However, their defects, including low sensitivity, delay of measurement results, need for calibration, poor comfort, and easy skin injury, are still important difficulties restricting their development. Moreover, the current research also lacks clinical trial data for a large sample population. In order to improve the sensitivity of glucose sensors at the electrochemical level, nanometer electrodes (such as nano-gold electrode, graphene or carbon nanotube electrode, etc.) are commonly used. Although the sensitivity of the sensor is improved, other problems such as high raw material cost and unsuitable mass production have followed. Within the scope of the optical, microwave and electrochemical methods discussed in this paper, all of these techniques estimate blood glucose levels indirectly rather than directly, so there is a difference between measured and actual blood glucose levels that requires subsequent correction. However, compared with optical and microwave methods, electrochemical methods have more advantages in the prospect of commercialization of non-invasive blood glucose. In the future, if a breakthrough can be made in the third-generation engineering enzyme and electrode materials for the non-invasive electrochemical sensor, and the problems of sensitivity, comfort and cost are solved, it can be expected that this will bring an unprecedented technological revolution and a wave of upgrades to the current glucose meter market.

In future research, if more physical parameters (such as pH, temperature, humidity, frequency) and other biomarkers associated with blood glucose can be combined to correct measurement results, to improve the accuracy of non-invasive skin glucose measurement and relevance to their blood glucose levels, and to achieve continuous monitoring of patients with hyperglycemia and hypoglycemia, this might be a more mainstream and feasible direction for the solution. All in all, there is a trend to integrate a series of functional modules on wearable devices in the future, which requires interdisciplinary and cross-direction cooperation between biomaterials, medicine, computer science, electrochemistry and other fields. In this way, a more robust and reliable, more sensitive and efficient, more portable and comfortable, more intelligent and modern non-invasive monitoring and closed-loop drug-delivery device can be developed to meet market expectations.

## Figures and Tables

**Table 1:** Saliva, tears, sweat and interstitial fluids (ISF)-based glucose monitoring devices comparison.

Sample	Advantages	Disadvantages	Electrode Materials	Sensitivity	Linear Range	Correlation Coefficient	LOD	Example
Saliva	Noninvasive; Easily collected	Many interfering impurities; Low correlation; Breeding bacteria; Low sensitivity; Hysteretic	SWNT-CS-GNp	26.6 μA mM^−1^	0.017~1.11 mM	0.995	--	Wenjun Zhang [108] (2015)
Tears	Noninvasive; Less impurity; interference; Wireless transmission	Low comfort; Energy supply problem; Poor precision; Hysteretic	PTB-GOx	0.421 μA mM^−1^	0.075~7.5 mM	0.9473	22.2 µM	Deonir Agustini [109] (2017)
Sweat	Noninvasive; Wearable; Easily collected; Continuous monitoring	Every analysis needs to sweat, not suitable for diabetics; Hysteretic	Nano-Gold	23.72 μA mM^−1^	0~1.4 mM	0.9951	10 μM	Qingfeng Zhai [110] (2019)
ISF	Noninvasive; Higher glucose concentration; Large linear rang; High sensitivity	Skin irritation Hysteretic	Nano-Gold	130.4 μA mM^−1^ 158.0 μA mM^−1^	0.005~0.035 mM 0.05~0.100 mM	>0.9	--	Yihao Chen [111] (2017)

## References

[1] Bruen D., Delaney C., Florea L., Diamond D. (2017). Glucose Sensing for Diabetes Monitoring: Recent Developments. Sensors.

[2] Gamble J.M., Clarke A., Myers K.J., Agnew M.D., Hatch K., Snow M.M., Davis E.M. (2015). Incretin-based medications for type 2 diabetes: An overview of reviews. Diabetes Obes. Metab..

[3] Nathan D.M., Group D.E.R. (2014). The diabetes control and complications trial/epidemiology of diabetes interventions and complications study at 30 years: Overview. Diabetes Care.

[4] Siddiqui S.A., Zhang Y., Lloret J., Song H., Obradovic Z. (2018). Pain-Free Blood Glucose Monitoring Using Wearable Sensors: Recent Advancements and Future Prospects. IEEE Rev. Biomed. Eng..

[5] Villena Gonzales W., Mobashsher A.T., Abbosh A. (2019). The Progress of Glucose Monitoring-A Review of Invasive to Minimally and Non-Invasive Techniques, Devices and Sensors. Sensors.

[6] International Diabetes Federation Guideline Development Group (2014). Global guideline for type 2 diabetes. Diabetes Res. Clin. Pract..

[7] Alberti K.G., Zimmet P., Shaw J. (2007). International Diabetes Federation: A consensus on Type 2 diabetes prevention. Diabet. Med..

[8] American Diabetes Association (2014). Standards of medical care in diabetes—2014. Diabetes Care.

[9] American Diabetes Association (2010). Standards of medical care in diabetes—2010. Diabetes Care.

[10] Nicholas D., Logan K.A., Sheng Y., Gao J., Farrell S., Dixon D., Callan B., McHale A.P., Callan J.F. (2018). Rapid paper based colorimetric detection of glucose using a hollow microneedle device. Int. J. Pharm..

[11] Teymourian H., Moonla C., Tehrani F., Vargas E., Aghavali R., Barfidokht A., Tangkuaram T., Mercier P.P., Dassau E., Wang J. (2020). Microneedle-Based Detection of Ketone Bodies along with Glucose and Lactate: Toward Real-Time Continuous Interstitial Fluid Monitoring of Diabetic Ketosis and Ketoacidosis. Anal. Chem..

[12] Bazaev N.A., Masloboev I.P., Selishchev S.V. (2011). Optical methods for noninvasive blood glucose monitoring. Med. Tekh..

[13] Takeuchi K., Kim B. (2018). Functionalized microneedles for continuous glucose monitoring. Nano Converg..

[14] Bollella P., Sharma S., Cass A.E.G., Tasca F., Antiochia R. (2019). Minimally Invasive Glucose Monitoring Using a Highly Porous Gold Microneedles-Based Biosensor: Characterization and Application in Artificial Interstitial Fluid. Catalysts.

[15] Kim K.B., Choi H., Jung H.J., Oh Y.J., Cho C.H., Min J.H., Yoon S., Kim J., Cho S.J., Cha H.J. (2019). Mussel-inspired enzyme immobilization and dual real-time compensation algorithms for durable and accurate continuous glucose monitoring. Biosens. Bioelectron..

[16] Ribet F., Stemme G., Roxhed N. (2018). Real-time intradermal continuous glucose monitoring using a minimally invasive microneedle-based system. Biomed. Microdevices.

[17] Clark L.C., Lyons C. (1962). Electrode systems for continuous monitoring in cardiovascular surgery. Ann. N. Y. Acad. Sci..

[18] Lee H., Hong Y.J., Baik S., Hyeon T., Kim D.H. (2018). Enzyme-Based Glucose Sensor: From Invasive to Wearable Device. Adv. Healthcare Mater..

[19] Oliver N.S., Toumazou C., Cass A.E., Johnston D.G. (2009). Glucose sensors: A review of current and emerging technology. Diabet. Med..

[20] Wang J. (2008). Electrochemical glucose biosensors. Chem. Rev..

[21] Yang H., Chung T.D., Kim Y.T., Choi C.A., Jun C.H., Kim H.C. (2002). Glucose sensor using a microfabricated electrode and electropolymerized bilayer films. Biosens. Bioelectron..

[22] Li J., Koinkar P., Fuchiwaki Y., Yasuzawa M. (2016). A fine pointed glucose oxidase immobilized electrode for low-invasive amperometric glucose monitoring. Biosens. Bioelectron..

[23] Wang H.C., Lee A.R. (2015). Recent developments in blood glucose sensors. J. Food Drug Anal..

[24] Lin M.J., Wu C.C., Chang K.S. (2019). Effect of Poly-l-Lysine Polycation on the Glucose Oxidase/Ferricyanide Composite-Based Second-Generation Blood Glucose Sensors. Sensors.

[25] Rabti A., Raouafi N., Merkoçi A. (2016). Bio(Sensing) devices based on ferrocene–functionalized graphene and carbon nanotubes. Carbon.

[26] Saleem M., Yu H., Wang L., Zain ul A., Khalid H., Akram M., Abbasi N.M., Huang J. (2015). Review on synthesis of ferrocene-based redox polymers and derivatives and their application in glucose sensing. Anal. Chim. Acta.

[27] Barathi P., Thirumalraj B., Chen S.-M., Angaiah S. (2019). A simple and flexible enzymatic glucose biosensor using chitosan entrapped mesoporous carbon nanocomposite. Microchem. J..

[28] Zhao L., Wen Z., Jiang F., Zheng Z., Lu S. (2020). Silk/polyols/GOD microneedle based electrochemical biosensor for continuous glucose monitoring. RSC Adv..

[29] Kim K.B., Lee W.-C., Cho C.-H., Park D.-S., Cho S.J., Shim Y.-B. (2019). Continuous glucose monitoring using a microneedle array sensor coupled with a wireless signal transmitter. Sens. Actuators B Chem..

[30] Bollella P., Sharma S., Cass A.E.G., Antiochia R. (2019). Minimally-invasive Microneedle-based Biosensor Array for Simultaneous Lactate and Glucose Monitoring in Artificial Interstitial Fluid. Electroanalysis.

[31] Wang Z., Li H., Wang J., Chen Z., Chen G., Wen D., Chan A., Gu Z. (2020). Transdermal colorimetric patch for hyperglycemia sensing in diabetic mice. Biomaterials.

[32] Chinnadayyala S.R., Park I., Cho S. (2018). Nonenzymatic determination of glucose at near neutral pH values based on the use of nafion and platinum black coated microneedle electrode array. Mikrochim. Acta.

[33] Sakudo A. (2016). Near-infrared spectroscopy for medical applications: Current status and future perspectives. Clin. Chim. Acta.

[34] Sari M.W., Luthfi M. Design and Analysis of Non-Invasive Blood Glucose Levels Monitoring. Proceedings of the 2016 1st International Seminar on Application for Technology of Information and Communication (Isemantic): Science and Technology for a Better Future.

[35] Kottmann J., Rey J.M., Sigrist M.W. (2016). Mid-Infrared Photoacoustic Detection of Glucose in Human Skin: Towards Non-Invasive Diagnostics. Sensors.

[36] Shokrekhodaei M., Quinones S. (2020). Review of Non-invasive Glucose Sensing Techniques: Optical, Electrical and Breath Acetone. Sensors.

[37] Singh K., Sandhu G.K., Lark B.S., Sud S.P. (2002). Molar extinction coefficients of some carbohydrates in aqueous solutions. Pramana J. Phys..

[38] Haxha S., Jhoja J. (2016). Optical Based Noninvasive Glucose Monitoring Sensor Prototype. IEEE Photonics J..

[39] Maruo K., Yamada Y. (2015). Near-infrared noninvasive blood glucose prediction without using multivariate analyses: Introduction of imaginary spectra due to scattering change in the skin. J. Biomed. Opt..

[40] Liu J., Liu R., Xu K. (2015). Accuracy of Noninvasive Glucose Sensing Based on Near-Infrared Spectroscopy. Appl. Spectrosc..

[41] Yadav J., Rani A., Singh V., Murari B.M. Near-infrared LED based Non-invasive Blood Glucose Sensor. Proceedings of the 2014 International Conference on Signal Processing and Integrated Networks (Spin).

[42] Rachim V.P., Chung W.-Y. (2019). Wearable-band type visible-near infrared optical biosensor for non-invasive blood glucose monitoring. Sens. Actuators B Chem..

[43] Aziz N.A.M., Arsad N., Menon P.S., Laili A.R., Laili M.H., Halim A.A.A. Analysis of Difference Light Sources for Non-Invasive Aqueous Glucose Detection. Proceedings of the 2014 IEEE 5th International Conference on Photonics (Icp).

[44] Pleitez M.A., Lieblein T., Bauer A., Hertzberg O., von Lilienfeld-Toal H., Mantele W. (2013). Windowless ultrasound photoacoustic cell for in vivo mid-IR spectroscopy of human epidermis: Low interference by changes of air pressure, temperature, and humidity caused by skin contact opens the possibility for a non-invasive monitoring of glucose in the interstitial fluid. Rev. Sci. Instrum..

[45] Zanon M., Riz M., Sparacino G., Facchinetti A., Suri R.E., Talary M.S., Cobelli C. (2011). Assessment of linear regression techniques for modeling multisensor data for non-invasive continuous glucose monitoring. Conf. Proc. IEEE Eng. Med. Biol. Soc..

[46] Jintao X., Liming Y., Yufei L., Chunyan L., Han C. (2017). Noninvasive and fast measurement of blood glucose in vivo by near infrared (NIR) spectroscopy. Spectrochim. Acta A Mol. Biomol. Spectrosc..

[47] Xue J., Ye L., Li C., Zhang M., Li P. (2018). Rapid and nondestructive measurement of glucose in a skin tissue phantom by near-infrared spectroscopy. Optik.

[48] Kuhner L., Semenyshyn R., Hentschel M., Neubrech F., Tarin C., Giessen H. (2019). Vibrational Sensing Using Infrared Nanoantennas: Toward the Noninvasive Quantitation of Physiological Levels of Glucose and Fructose. ACS Sens..

[49] Mazarevica G., Freivalds T., Jurka A. (2002). Properties of erythrocyte light refraction in diabetic patients. J. Biomed. Opt..

[50] Maruo K., Tsurugi M., Jakusei C., Ota T., Arimoto H., Yamada Y., Tamura M., Ishii M., Ozaki Y. (2003). Noninvasive blood glucose assay using a newly developed near-infrared system. IEEE J. Sel. Top. Quantum Electron..

[51] Sim J.Y., Ahn C.G., Jeong E.J., Kim B.K. (2018). In vivo Microscopic Photoacoustic Spectroscopy for Non-Invasive Glucose Monitoring Invulnerable to Skin Secretion Products. Sci. Rep..

[52] Kasahara R., Kino S., Soyama S., Matsuura Y. (2018). Unsupervised calibration for noninvasive glucose-monitoring devices using mid-infrared spectroscopy. J. Innov. Opt. Health Sci..

[53] Kasahara R., Kino S., Soyama S., Matsuura Y. (2018). Noninvasive glucose monitoring using mid-infrared absorption spectroscopy based on a few wavenumbers. Biomed. Opt. Express.

[54] Zirk K., Poetzschke H. (2004). On the suitability of refractometry for the analysis of glucose in blood-derived fluids. Med. Eng. Phys..

[55] Pravdin A.B., Spivak V.A., Yakovlev D.A. (2016). On the possibility of noninvasive polarimetric determination of glucose content in skin. Opt. Spectrosc..

[56] Purvinis G., Cameron B.D., Altrogge D.M. (2011). Noninvasive polarimetric-based glucose monitoring: An in vivo study. J. Diabetes Sci. Technol..

[57] Malik B.H., Cote G.L. (2010). Characterizing dual wavelength polarimetry through the eye for monitoring glucose. Biomed. Opt. Express.

[58] Malik B.H., Cote G.L. (2010). Modeling the corneal birefringence of the eye toward the development of a polarimetric glucose sensor. J. Biomed. Opt..

[59] McNichols R.J., Cote G.L. (2000). Optical glucose sensing in biological fluids: An overview. J. Biomed. Opt..

[60] Menguc E., Helhel S. (2019). Relationship between Human Glucose Level and Optical De/Polarization Information in 600 nm–800 nm Wavelength Region. Photonlcs Electromagn. Res. Symp..

[61] Yadav J., Rani A., Singh V., Murari B.M. (2015). Comparative Study of Different Measurement Sites Using NIR Based Non-invasive Glucose Measurement System. Procedia Comput. Sci..

[62] Lo Y.-L., Yu T.-C. (2006). A polarimetric glucose sensor using a liquid-crystal polarization modulator driven by a sinusoidal signal. Opt. Commun..

[63] Guo X., Wood M.F.G., Vitkin I.A. (2006). Angular measurements of light scattered by turbid chiral media using linear Stokes polarimeter. J. Biomed. Opt..

[64] Ansari R.R., Böckle S., Rovati L. (2004). New optical scheme for a polarimetric-based glucose sensor. J. Biomed. Opt..

[65] Pirnstill C.W., Malik B.H., Gresham V.C., Cote G.L. (2012). In vivo glucose monitoring using dual-wavelength polarimetry to overcome corneal birefringence in the presence of motion. Diabetes Technol. Ther..

[66] Stark C., Behroozian R., Redmer B., Fiedler F., Muller S. (2019). Real-time compensation method for robust polarimetric determination of glucose in turbid media. Biomed. Opt. Express.

[67] Malik B.H., Pirnstill C.W., Cote G.L. (2013). Dual-wavelength polarimetric glucose sensing in the presence of birefringence and motion artifact using anterior chamber of the eye phantoms. J. Biomed. Opt..

[68] Tang Z., Barton S.J., Ward T.E., Lowry J.P., Doran M.M., Byrne H.J., Hennelly B.M. (2018). Multicomponent analysis using a confocal Raman microscope. Appl. Opt..

[69] Yang D., Afroosheh S., Lee J.O., Cho H., Kumar S., Siddique R.H., Narasimhan V., Yoon Y.Z., Zayak A.T., Choo H. (2018). Glucose Sensing Using Surface-Enhanced Raman-Mode Constraining. Anal. Chem..

[70] Singh S.P., Mukherjee S., Galindo L.H., So P.T.C., Dasari R.R., Khan U.Z., Kannan R., Upendran A., Kang J.W. (2018). Evaluation of accuracy dependence of Raman spectroscopic models on the ratio of calibration and validation points for non-invasive glucose sensing. Anal. Bioanal. Chem..

[71] Shao J., Lin M., Li Y., Li X., Liu J., Liang J., Yao H. (2012). In vivo blood glucose quantification using Raman spectroscopy. PLoS ONE.

[72] Li G., Zhou M., Wu H.J., Lin L. (2010). The Research Status and Development of Noninvasive Glucose Optical Measurements. Spectrosc. Spect. Anal..

[73] Cho M.-J., Park S.-Y. (2019). Carbon-dot-based ratiometric fluorescence glucose biosensor. Sens. Actuators B Chem..

[74] Zhai H., Bai Y., Wang H., Qin J., Liu H., Feng F. (2018). Development of a novel fluorescence ratiometric glucose sensor based on carbon dots and a potential fluorophore m-dihydroxybenzene. Anal. Methods.

[75] Zhai H., Bai Y., Qin J., Feng F. (2019). Colorimetric and Ratiometric Fluorescence Dual-Mode Sensing of Glucose Based on Carbon Quantum Dots and Potential UV/Fluorescence of o-Diaminobenzene. Sensors.

[76] Chen L., Tse W.H., Chen Y., McDonald M.W., Melling J., Zhang J. (2017). Nanostructured biosensor for detecting glucose in tear by applying fluorescence resonance energy transfer quenching mechanism. Biosens. Bioelectron..

[77] Lan Y.T., Kuang Y.P., Zhou L.P., Wu G.Y., Gu P.C., Wei H.J., Chen K. (2017). Noninvasive monitoring of blood glucose concentration in diabetic patients with optical coherence tomography. Laser Phys. Lett..

[78] Larin K.V., Tuchin V.V. (2008). Functional imaging and assessment of the glucose diffusion rate in epithelial tissues in optical coherence tomography. Quantum Electron..

[79] Chen T.L., Lo Y.L., Liao C.C., Phan Q.H. (2018). Noninvasive measurement of glucose concentration on human fingertip by optical coherence tomography. J. Biomed. Opt..

[80] De Pretto L.R., Yoshimura T.M., Ribeiro M.S., Zanardi de Freitas A. (2016). Optical coherence tomography for blood glucose monitoring in vitro through spatial and temporal approaches. J. Biomed. Opt..

[81] Yilmaz T., Foster R., Hao Y. (2019). Radio-Frequency and Microwave Techniques for Non-Invasive Measurement of Blood Glucose Levels. Diagnostics.

[82] Zhang R., Liu S., Jin H., Luo Y., Zheng Z., Gao F., Zheng Y. (2019). Noninvasive Electromagnetic Wave Sensing of Glucose. Sensors.

[83] Turgul V., Kale I. (2017). Simulating the Effects of Skin Thickness and Fingerprints to Highlight Problems with Non-Invasive RF Blood Glucose Sensing From Fingertips. IEEE Sens. J..

[84] Hofmann M., Fischer G., Weigel R., Kissinger D. (2013). Microwave-Based Noninvasive Concentration Measurements for Biomedical Applications. IEEE Trans. Microw. Theory Tech..

[85] Choi H., Luzio S., Beutler J., Porch A. Microwave Noninvasive Blood Glucose Monitoring Sensor: Penetration Depth and Sensitivity Analysis. Proceedings of the 2018 Ieee/Mtt-S International Microwave Biomedical Conference (Imbioc).

[86] Gabriel S., Lau R.W., Gabriel C. (1996). The dielectric properties of biological tissues: III. Parametric models for the dielectric spectrum of tissues. Phys. Med. Biol..

[87] Jang C., Park J.-K., Lee H.-J., Yun G.-H., Yook J.-G. (2020). Non-Invasive Fluidic Glucose Detection Based on Dual Microwave Complementary Split Ring Resonators with a Switching Circuit for Environmental Effect Elimination. IEEE Sens. J..

[88] Choi H., Naylon J., Luzio S., Beutler J., Birchall J., Martin C., Porch A. (2015). Design and In Vitro Interference Test of Microwave Noninvasive Blood Glucose Monitoring Sensor. IEEE Trans. Microw. Theory Tech..

[89] Yilmaz T., Foster R., Hao Y. (2014). Towards Accurate Dielectric Property Retrieval of Biological Tissues for Blood Glucose Monitoring. IEEE Trans. Microw. Theory Tech..

[90] Omer A.E., Gigoyan S., Shaker G., Safavi-Naeini S. (2020). WGM-Based Sensing of Characterized Glucose- Aqueous Solutions at mm-Waves. IEEE Access.

[91] Kumar A., Wang C., Meng F.Y., Zhou Z.L., Zhao M., Yan G.F., Kim E.S., Kim N.Y. (2020). High-Sensitivity, Quantified, Linear and Mediator-Free Resonator-Based Microwave Biosensor for Glucose Detection. Sensors.

[92] Satish S.K., Anand S. (2020). Demonstration of Microstrip Sensor for the Feasibility Study of Non-invasive Blood-Glucose Sensing. Mapan.

[93] Omer A.E., Safavi-Naeini S., Hughson R., Shaker G. (2020). Blood Glucose Level Monitoring Using an FMCW Millimeter-Wave Radar Sensor. Remote Sens..

[94] Xiao X., Li Q. (2017). A Noninvasive Measurement of Blood Glucose Concentration by UWB Microwave Spectrum. IEEE Antennas Wirel. Propag. Lett..

[95] Vrba J., Karch J., Vrba D. (2015). Phantoms for Development of Microwave Sensors for Noninvasive Blood Glucose Monitoring. Int. J. Antennas Propag..

[96] Kim S., Melikyan H., Kim J., Babajanyan A., Lee J.H., Enkhtur L., Friedman B., Lee K. (2012). Noninvasive in vitro measurement of pig-blood d-glucose by using a microwave cavity sensor. Diabetes Res. Clin. Pract..

[97] Garg S.K., Potts R.O., Ackerman N.R., Fermi S.J., Tamada J.A., Chase H.P. (1999). Correlation at fingerstick blood glucose measurements with GlucoWatch biographer glucose results in young subjects with type 1 diabetes. Diabetes Care.

[98] Tierney M.J., Tamada J.A., Potts R.O., Jovanovic L., Garg S., Team C.R. (2001). Clinical evaluation of the GlucoWatch (R) biographer: A continual, non-invasive glucose monitor for patients with diabetes. Biosens. Bioelectron..

[99] Potts R.O., Tamada A.J., Tierney M.J. (2002). Glucose monitoring by reverse iontophoresis. Diabetes/Metab. Res. Rev..

[100] Giri T.K., Chakrabarty S., Ghosh B. (2017). Transdermal reverse iontophoresis: A novel technique for therapeutic drug monitoring. J. Control Release.

[101] Bandodkar A.J., Jia W., Yardimci C., Wang X., Ramirez J., Wang J. (2015). Tattoo-based noninvasive glucose monitoring: A proof-of-concept study. Anal. Chem..

[102] Pandey P.C., Shukla S., Skoog S.A., Boehm R.D., Narayan R.J. (2019). Current Advancements in Transdermal Biosensing and Targeted Drug Delivery. Sensors.

[103] Vashist S.K. (2012). Non-invasive glucose monitoring technology in diabetes management: A review. Anal. Chim. Acta.

[104] Zhao R., Wang C., Lu F., Du L., Fang Z., Guo X., Liu J.T., Chen C.J., Zhao Z. (2018). A Flexible Interdigital Electrode Used in Skin Penetration Promotion and Evaluation with Electroporation and Reverse Iontophoresis Synergistically. Sensors.

[105] Mandal S., Marie M., Kuchuk A., Manasreh M.O., Benamara M. (2016). Sensitivity enhancement in an in-vitro glucose sensor using gold nanoelectrode ensembles. J. Mater. Sci. Mater. Electron..

[106] Claussen J.C., Wickner M.M., Fisher T.S., Porterfield D.M. (2011). Transforming the fabrication and biofunctionalization of gold nanoelectrode arrays into versatile electrochemical glucose biosensors. ACS Appl. Mater. Interfaces.

[107] Lantiat D., Vivier V., Laberty-Robert C., Grosso D., Sanchez C. (2010). Gold nanoelectrode arrays and their evaluation by impedance spectroscopy and cyclic voltammetry. Chemphyschem.

[108] Zhang W., Du Y., Wang M.L. (2015). Noninvasive glucose monitoring using saliva nano-biosensor. Sens. Bio-Sens. Res..

[109] Agustini D., Bergamini M.F., Marcolino-Junior L.H. (2017). Tear glucose detection combining microfluidic thread based device, amperometric biosensor and microflow injection analysis. Biosens. Bioelectron..

[110] Zhai Q., Gong S., Wang Y., Lyu Q., Liu Y., Ling Y., Wang J., Simon G.P., Cheng W. (2019). Enokitake Mushroom-like Standing Gold Nanowires toward Wearable Noninvasive Bimodal Glucose and Strain Sensing. ACS Appl. Mater. Interfaces.

[111] Chen Y.H., Lu S.Y., Zhang S.S., Li Y., Qu Z., Chen Y., Lu B.W., Wang X.Y., Feng X. (2017). Skin-like biosensor system via electrochemical channels for noninvasive blood glucose monitoring. Sci. Adv..

[112] Malon R.S., Sadir S., Balakrishnan M., Corcoles E.P. (2014). Saliva-based biosensors: Noninvasive monitoring tool for clinical diagnostics. BioMed Res. Int..

[113] Naseri R., Mozaffari H.R., Ramezani M., Sadeghi M. (2018). Effect of diabetes mellitus type 2 on salivary glucose, immunoglobulin A, total protein, and amylase levels in adults: A systematic review and meta-analysis of case-control studies. J. Res. Med. Sci..

[114] Yang Y., Gao W. (2019). Wearable and flexible electronics for continuous molecular monitoring. Chem. Soc. Rev..

[115] Heikenfeld J., Jajack A., Feldman B., Granger S.W., Gaitonde S., Begtrup G., Katchman B.A. (2019). Accessing analytes in biofluids for peripheral biochemical monitoring. Nat. Biotechnol..

[116] Mathew T.L., Pownraj P., Abdulla S., Pullithadathil B. (2015). Technologies for Clinical Diagnosis Using Expired Human Breath Analysis. Diagnostics.

[117] Wang Z., Wang C. (2013). Is breath acetone a biomarker of diabetes? A historical review on breath acetone measurements. J. Breath Res..

[118] Masikini M., Chowdhury M., Nemraoui O. (2020). Review—Metal Oxides: Application in Exhaled Breath Acetone Chemiresistive Sensors. J. Electrochem. Soc..

[119] Wang C., Mbi A., Shepherd M. (2010). A Study on Breath Acetone in Diabetic Patients Using a Cavity Ringdown Breath Analyzer: Exploring Correlations of Breath Acetone With Blood Glucose and Glycohemoglobin A1C. IEEE Sens. J..

[120] Turner C., Walton C., Hoashi S., Evans M. (2009). Breath acetone concentration decreases with blood glucose concentration in type I diabetes mellitus patients during hypoglycaemic clamps. J. Breath Res..

[121] Rydosz A. (2015). A Negative Correlation Between Blood Glucose and Acetone Measured in Healthy and Type 1 Diabetes Mellitus Patient Breath. J. Diabetes Sci. Technol..

[122] Wilson A.D. (2015). Advances in electronic-nose technologies for the detection of volatile biomarker metabolites in the human breath. Metabolites.

[123] Sun M., Chen Z., Gong Z., Zhao X., Jiang C., Yuan Y., Wang Z., Li Y., Wang C. (2015). Determination of breath acetone in 149 Type 2 diabetic patients using a ringdown breath-acetone analyzer. Anal. Bioanal. Chem..

[124] Lavanya N., Leonardi S.G., Marini S., Espro C., Kanagaraj M., Reddy S.L., Sekar C., Neri G. (2020). MgNi_2_O_3_ nanoparticles as novel and versatile sensing material for non-enzymatic electrochemical sensing of glucose and conductometric determination of acetone. J. Alloys Compd..

[125] Saasa V., Beukes M., Lemmer Y., Mwakikunga B. (2019). Blood Ketone Bodies and Breath Acetone Analysis and Their Correlations in Type 2 Diabetes Mellitus. Diagnostics.

[126] Behera B., Joshi R., Anil Vishnu G.K., Bhalerao S., Pandya H.J. (2019). Electronic nose: A non-invasive technology for breath analysis of diabetes and lung cancer patients. J. Breath Res..

[127] Liu T., Li L., Yang X., Liang X., Liu F., Liu F., Zhang C., Sun P., Yan X., Lu G. (2019). Mixed potential type acetone sensor based on Ce_0.8_Gd_0.2_O_1.95_ and Bi_0.5_La_0.5_FeO_3_ sensing electrode used for the detection of diabetic ketosis. Sens. Actuators B Chem..

[128] Rabih A.A.S., Dennis J.O., Ahmed A.Y., Md Khir M.H., Ahmed M.G.A., Idris A., Mian M.U. (2018). MEMS-Based Acetone Vapor Sensor for Non-Invasive Screening of Diabetes. IEEE Sens. J..

[129] Mansouri S., Boulares S., Alhadidi T. (2020). Non-invasive Measurement of Blood Glucose by Breath Analysis. IEEJ Trans. Electr. Electron. Eng..

[130] Badugu R., Reece E.A., Lakowicz J.R. (2018). Glucose-sensitive silicone hydrogel contact lens toward tear glucose monitoring. J. Biomed. Opt..

[131] Gabriel E., Garcia P., Lopes F., Coltro W. (2017). Paper-Based Colorimetric Biosensor for Tear Glucose Measurements. Micromachines.

[132] Kownacka A.E., Vegelyte D., Joosse M., Anton N., Toebes B.J., Lauko J., Buzzacchera I., Lipinska K., Wilson D.A., Geelhoed-Duijvestijn N. (2018). Clinical Evidence for Use of a Noninvasive Biosensor for Tear Glucose as an Alternative to Painful Finger-Prick for Diabetes Management Utilizing a Biopolymer Coating. Biomacromolecules.

[133] Reid R.C., Minteer S.D., Gale B.K. (2015). Contact lens biofuel cell tested in a synthetic tear solution. Biosens. Bioelectron..

[134] La Belle J.T., Adams A., Lin C.-E., Engelschall E., Pratt B., Cook C.B. (2016). Self-monitoring of tear glucose: The development of a tear based glucose sensor as an alternative to self-monitoring of blood glucose. Chem. Commun..

[135] Chu M.X., Miyajima K., Takahashi D., Arakawa T., Sano K., Sawada S., Kudo H., Iwasaki Y., Akiyoshi K., Mochizuki M. (2011). Soft contact lens biosensor for in situ monitoring of tear glucose as non-invasive blood sugar assessment. Talanta.

[136] Sealy C. (2018). Glucose monitoring in sweat and tears no stretch for new biosensors. Nano Today.

[137] Park J., Kim J., Kim S.Y., Cheong W.H., Jang J., Park Y.G., Na K., Kim Y.T., Heo J.H., Lee C.Y. (2018). Soft, smart contact lenses with integrations of wireless circuits, glucose sensors, and displays. Sci. Adv..

[138] Lee H., Song C., Hong Y.S., Kim M.S., Cho H.R., Kang T., Shin K., Choi S.H., Hyeon T., Kim D.H. (2017). Wearable/disposable sweat-based glucose monitoring device with multistage transdermal drug delivery module. Sci. Adv..

[139] Gao W., Ota H., Kiriya D., Takei K., Javey A. (2019). Flexible Electronics toward Wearable Sensing. Acc. Chem. Res..

[140] Bandodkar A.J., Gutruf P., Choi J., Lee K., Sekine Y., Reeder J.T., Jeang W.J., Aranyosi A.J., Lee S.P., Model J.B. (2019). Battery-free, skin-interfaced microfluidic/electronic systems for simultaneous electrochemical, colorimetric, and volumetric analysis of sweat. Sci. Adv..

[141] Kim J., Sempionatto J.R., Imani S., Hartel M.C., Barfidokht A., Tang G., Campbell A.S., Mercier P.P., Wang J. (2018). Simultaneous Monitoring of Sweat and Interstitial Fluid Using a Single Wearable Biosensor Platform. Adv. Sci. (Weinh).

[142] Lipani L., Dupont B.G.R., Doungmene F., Marken F., Tyrrell R.M., Guy R.H., Ilie A. (2018). Non-invasive, transdermal, path-selective and specific glucose monitoring via a graphene-based platform. Nat. Nanotechnol..

[143] Sabri A.H., Kim Y., Marlow M., Scurr D.J., Segal J., Banga A.K., Kagan L., Lee J.B. (2019). Intradermal and transdermal drug delivery using microneedles-Fabrication, performance evaluation and application to lymphatic delivery. Adv. Drug Deliv. Rev..

[144] Ronnander P., Simon L., Koch A. (2020). Experimental and mathematical study of the transdermal delivery of sumatriptan succinate from polyvinylpyrrolidone-based microneedles. Eur. J. Pharm. Biopharm..

[145] Yang J., Liu X., Fu Y., Song Y. (2019). Recent advances of microneedles for biomedical applications: Drug delivery and beyond. Acta Pharm. Sin. B.

[146] Waghule T., Singhvi G., Dubey S.K., Pandey M.M., Gupta G., Singh M., Dua K. (2019). Microneedles: A smart approach and increasing potential for transdermal drug delivery system. Biomed. Pharmacother..

[147] Zhu J., Zhou X., Kim H.J., Qu M., Jiang X., Lee K., Ren L., Wu Q., Wang C., Zhu X. (2020). Gelatin Methacryloyl Microneedle Patches for Minimally Invasive Extraction of Skin Interstitial Fluid. Small.

